# Innate immune activation restricts priming and protective efficacy of the radiation-attenuated PfSPZ malaria vaccine

**DOI:** 10.1172/jci.insight.167408

**Published:** 2024-04-30

**Authors:** Leetah Senkpeil, Jyoti Bhardwaj, Morgan R. Little, Prasida Holla, Aditi Upadhye, Elizabeth M. Fusco, Phillip A. Swanson, Ryan E. Wiegand, Michael D. Macklin, Kevin Bi, Barbara J. Flynn, Ayako Yamamoto, Erik L. Gaskin, D. Noah Sather, Adrian L. Oblak, Edward Simpson, Hongyu Gao, W. Nicholas Haining, Kathleen B. Yates, Xiaowen Liu, Tooba Murshedkar, Thomas L. Richie, B. Kim Lee Sim, Kephas Otieno, Simon Kariuki, Xiaoling Xuei, Yunlong Liu, Rafael B. Polidoro, Stephen L. Hoffman, Martina Oneko, Laura C. Steinhardt, Nathan W. Schmidt, Robert A. Seder, Tuan M. Tran

**Affiliations:** 1 Division of Infectious Diseases, Department of Medicine,; 2 Department of Microbiology and Immunology, and; 3 Ryan White Center for Pediatric Infectious Diseases and Global Health, Department of Pediatrics, Indiana University School of Medicine, Indianapolis, Indiana, USA.; 4 Cellular Immunology Section, Vaccine Research Center, National Institute of Allergy and Infectious Diseases (NIAID), NIH, Bethesda, Maryland, USA.; 5 Malaria Branch, Division of Parasitic Diseases and Malaria, Center for Global Health, Centers for Disease Control and Prevention, Atlanta, Georgia, USA.; 6 Broad Institute of MIT and Harvard, Cambridge, Massachusetts, USA.; 7 Center for Global Infectious Disease Research, Seattle Children’s Research Institute, Seattle, Washington, USA.; 8 Stark Neurosciences Research Institute and; 9 Center for Medical Genomics, Indiana University School of Medicine, Indianapolis, Indiana, USA.; 10 Department of Pediatric Oncology, Dana-Farber Cancer Institute, Boston, Massachusetts, USA.; 11 Deming Department of Medicine, Tulane University School of Medicine, New Orleans, Louisiana, USA.; 12 Sanaria, Rockville, Maryland, USA.; 13 Kenya Medical Research Institute, Centre for Global Health Research, Kisumu, Kenya.

**Keywords:** Infectious disease, Vaccines, Adaptive immunity, Innate immunity, Malaria

## Abstract

A systems analysis was conducted to determine the potential molecular mechanisms underlying differential immunogenicity and protective efficacy results of a clinical trial of the radiation-attenuated whole-sporozoite PfSPZ vaccine in African infants. Innate immune activation and myeloid signatures at prevaccination baseline correlated with protection from *P. falciparum* parasitemia in placebo controls. These same signatures were associated with susceptibility to parasitemia among infants who received the highest and most protective PfSPZ vaccine dose. Machine learning identified spliceosome, proteosome, and resting DC signatures as prevaccination features predictive of protection after highest-dose PfSPZ vaccination, whereas baseline circumsporozoite protein–specific (CSP-specific) IgG predicted nonprotection. Prevaccination innate inflammatory and myeloid signatures were associated with higher sporozoite-specific IgG Ab response but undetectable PfSPZ-specific CD8^+^ T cell responses after vaccination. Consistent with these human data, innate stimulation in vivo conferred protection against infection by sporozoite injection in malaria-naive mice while diminishing the CD8^+^ T cell response to radiation-attenuated sporozoites. These data suggest a dichotomous role of innate stimulation for malaria protection and induction of protective immunity by whole-sporozoite malaria vaccines. The uncoupling of vaccine-induced protective immunity achieved by Abs from more protective CD8^+^ T cell responses suggests that PfSPZ vaccine efficacy in malaria-endemic settings may be constrained by opposing antigen presentation pathways.

## Abstract 2

A systems analysis of a clinical trial in African infants reveals prevaccination signatures predictive of immunogenicity and efficacy of a radiation-attenuated whole sporozoite malaria vaccine.

## Graphical abstract

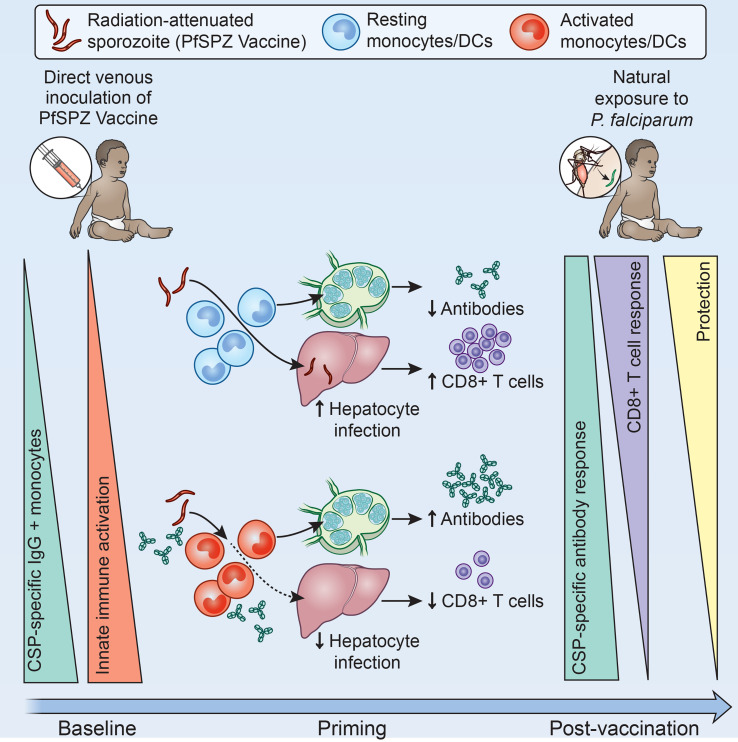

## Introduction

Preerythrocytic malaria vaccines target the *Plasmodium* parasite prior to the symptomatic blood stage and aim to induce sterilizing immunity that inhibits sporozoite entry into hepatocytes or impedes liver-stage development. The most advanced malaria vaccines, RTS,S and R21, are subunit vaccines composed of an immunodominant B cell epitope (asparagine-alanine-asparagine-proline [NANP] repeats) and T cell epitopes from the *P. falciparum* (Pf) circumsporozoite protein (CSP) fused to hepatitis B surface antigen. In a phase III trial of infants in sub-Saharan Africa, a 4-dose RTS,S/AS01 regimen conferred 36.3% vaccine efficacy (VE) against clinical malaria over 4 years (1). A next-generation RTS,S-like vaccine, R21, demonstrated 77% VE against clinical malaria over a 6-month malaria season in infants (2), with 80% efficacy during the subsequent season following a booster (3). Immunization with radiation-attenuated sporozoites (RAS) represents another approach for inducing protection against *Plasmodium* infection. Initially demonstrated in mice (4), RAS immunization was later shown to be protective in humans (5). Induction of sterilizing immunity requires that RAS undergo arrested intrahepatocytic development after liver infection (6). Aseptic, purified, live, nonreplicating, radiation-attenuated cryopreserved Pf sporozoites (PfSPZ vaccine) developed for direct venous inoculation (DVI) showed approximately 60%–100% VE against parasitemia up to 14 months after challenge by controlled human malaria infection (CHMI) with homologous parasites in malaria-naive adults (7). When delivered via DVI, the PfSPZ vaccine confers protection by inducing CSP-specific Abs and both peripheral and liver-resident Pf-specific T cell responses (7–9), with the latter critical for durable sterilizing immunity (10, 11). Furthermore, γδ (Vγ9Vδ2) T cells expand with PfSPZ vaccination, and their preimmunization frequencies correlate with vaccine-induced Pf-specific T cells and protection, perhaps by priming protective T cell responses (9, 12).

**Figure 1:**
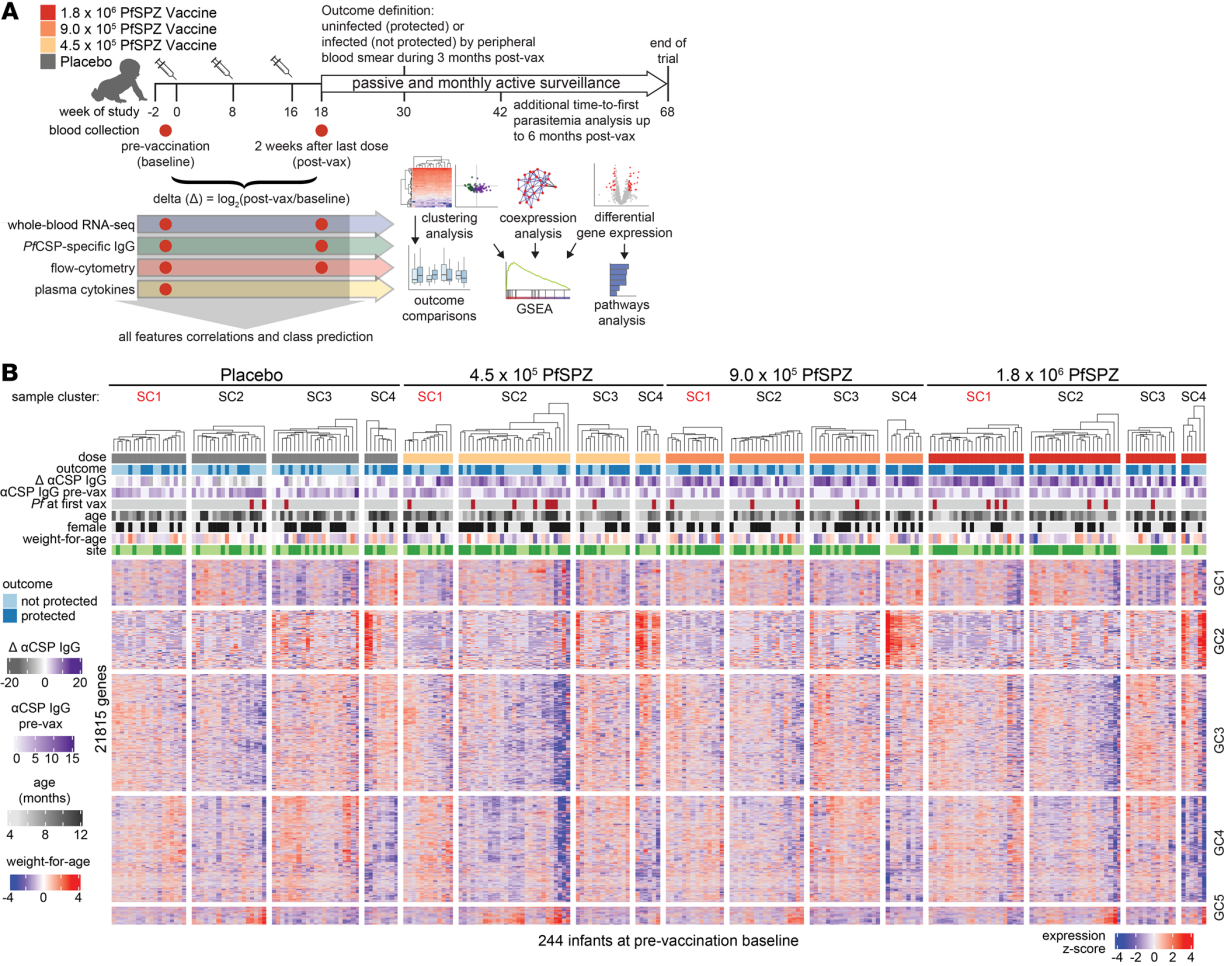
Variation in baseline transcriptomes. (**A**) Overall study design. (**B**) Clustering heatmap of baseline transcriptomes. Partitioning around medoids and Euclidean distance metric were used for clustering with *k* = 4 and *k* = 5 for sample clusters (SC) and gene clusters (GC), respectively. Samples (columns) were split by treatment to highlight the patterns within and between treatments.

Identical PfSPZ vaccine regimens were less immunogenic and effective in trials of malaria-exposed African adults when compared with malaria-naive adults (9, 13, 14), suggesting that malaria exposure may limit vaccine immunogenicity and efficacy. Infants in sub-Saharan Africa are relatively malaria inexperienced but have the greatest risk for severe malaria and death. Based on the hypothesis that less prior malaria exposure may enhance vaccine responsiveness, a randomized, placebo-controlled trial of the PfSPZ vaccine was conducted in Kenyan infants (15). Groups of 84 infants received 4.5 × 10^5^, 9.0 × 10^5^, or 1.8 × 10^6^ PfSPZ vaccine or normal saline (placebo) 3 times at 8-week intervals (Figure 1A). Although there was no significant VE against Pf parasitemia for any dose group at the primary endpoint of 6 months after immunization, VE in the highest dose group was 41.1% at 3 months. PfSPZ vaccination generated robust CSP-specific Abs that modestly correlated with protection but low or undetectable Pf-specific T cell responses (15). The relatively balanced outcomes provided an opportunity to investigate the molecular differences between infants who did and did not effectively respond to the PfSPZ vaccine as measured by immunogenicity or protection against parasitemia. To gain better insight into the mechanisms underlying the immunogenicity and efficacy results from this trial, we used pre- and postvaccination blood samples to conduct a systems analysis that integrated whole-blood transcriptomic profiling with CSP-specific Ab, immunophenotyping, plasma cytokine, and clinical data (Figure 1A and Supplemental Figure 1; supplemental material available online with this article; https://doi.org/10.1172/jci.insight.167408DS1).

## Results

### Clinical outcomes and overview of baseline transcriptomes.

In the clinical trial, the primary outcome was presence (not protected; NP) or absence (protected; P) of microscopy-detectable Pf parasitemia through 6 months of surveillance after immunization (15). Here, we used the secondary outcomes of protection through 3 months or days to first parasitemia up to 168 days (Figure 1A). Among 336 participants, 258 had whole-blood RNA from at least 1 time point (Supplemental Figure 1A). Characteristics between outcomes by dose were similar except for differences in malaria transmission between study sites (Supplemental Figure 1B). Samples were evenly distributed across groups (Supplemental Figure 1C). To determine whether baseline transcriptomes can distinguish NP from P infants, we performed unsupervised clustering analysis of prevaccination blood transcriptomes from 244 infants (Figure 1, A and B). A sample cluster (SC1) within the 1.8 × 10^6^ PfSPZ group was significantly overrepresented by P infants (Figure 1B and Supplemental Figure 2, A and B). No significant differences were observed between P and NP within either SC1 or SC2-4 for any dose group for CSP-specific IgG, weight-for-age, age, sex, parasitemia at first vaccination, or study site (Supplemental Figure 2, C–I). This suggests that a prevaccination transcriptomic signature may be able to distinguish outcomes after high-dose PfSPZ vaccination independently of Ab responses or potential confounders of malaria risk.

### Innate activation, myeloid, and erythroid signatures at baseline distinguish protective outcomes.

**Figure 2:**
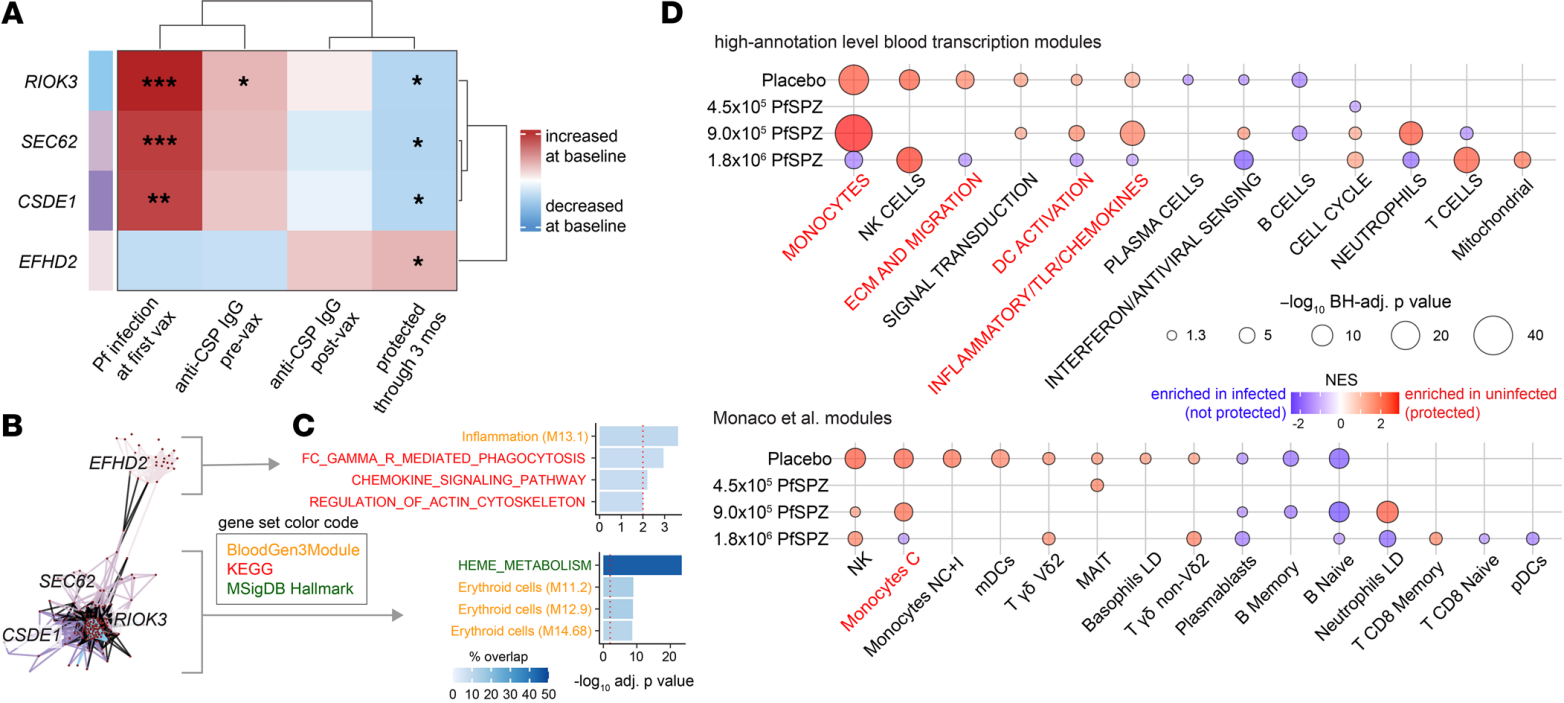
Innate activation, myeloid, and erythroid signatures at baseline distinguish protective outcomes. (**A**) Associations between module eigengenes, obtained by weighted gene correlation network analysis using baseline transcriptomes of all 244 infants, with indicated binary variables determined by empirical Bayes moderated *t* test (*P < 0.05, **P < 0.01, ***P < 0.001). (**B**) Network graphs of significant modules containing nodes (red dots), edges (lines), and intermodule correlations (black edges). (**C**) Overrepresentation analysis of modules significantly correlating with outcome. Genes within the highly interconnected modules *CSDE2*, *RIOK3*, and *SEC62* were combined. Only pathways/modules with BH-adjusted *P* < 0.01 are shown. (**D**) Gene set enrichment analysis between P and NP infants by group. Only modules with a BH-adjusted *P* < 0.05 are shown. Red text are modules in which direction of normalized enrichment score (NES) is reversed between placebo and 1.8 × 10^6^ PfSPZ. BH, Benjamini-Hochberg.

To determine the relationship between genes with highly correlated expression at baseline and protective outcomes irrespective of vaccination, we constructed data-driven modules from the 244 prevaccination transcriptomes using weighted gene coexpression network analysis (WGCNA) and correlated these to parasitemia and CSP-specific IgG variables. Three modules led by the hub genes *RIOK3*, *CSDE1*, and *SEC62* negatively associated with protection and positively associated with both prevaccination anti–CSP IgG and parasitemia at first vaccination (Figure 2A and Supplemental Figure 3A). One module positively correlated with protection and was led by the hub gene *EFHD2*, which encodes swiprosin-1, a calcium-binding protein involved in the macrophage response to sepsis (16). Network graphs of nodal correlations reveal that modules negatively associated with protection were tightly linked to each other but weakly linked to the protection-associated *EFHD2* module (Figure 2B). The *EFHD2* network was overrepresented by genes related to inflammation, Fcγ receptor–mediated phagocytosis, and chemokine signaling. By contrast, the network composed of modules negatively associated with protection was enriched in genes related to heme metabolism and erythrocytes (Figure 2C).

We next compared baseline transcriptomes between the 3-month outcomes for each dose group using differential gene expression (DGE) analysis adjusting for batch, gender, site, baseline CSP-specific IgG, and parasitemia at first vaccination. No differentially expressed genes were observed between outcomes for any dose group at FDR < 5% (Supplemental Table 1). However, gene set enrichment analysis (GSEA) revealed significant transcriptomic differences between P and NP infants at FDR < 5%, especially in placebo, 9.0 × 10^5^ PfSPZ, and 1.8 × 10^5^ PfSPZ (Figure 2D and Supplemental Figure 3B). Enrichment direction was similar between 9.0 × 10^5^ PfSPZ and placebo for mutually enriched modules, consistent with the statistical lack of protection for 9.0 × 10^5^ PfSPZ. By contrast, multiple modules were differentially enriched in a reciprocal manner between 1.8 × 10^6^ PfSPZ and placebo. Myeloid (monocytes and “DC activation”) and innate inflammatory (“inflammatory/TLR/chemokines”, “TNF via NFκB signaling”) signatures were enriched in P versus NP for placebo but were conversely enriched in NP versus P for 1.8 × 10^6^ PfSPZ, suggesting the presence of these baseline signatures had opposite effects on natural and vaccine-induced protection. Other baseline signatures that were differentially enriched in a reciprocal manner between placebo and 1.8 × 10^6^ PfSPZ include “extracellular matrix (ECM) and migration”, “heme metabolism”, “platelet/prostaglandins”, and “erythroid cells”. These data suggest that prevaccination innate immune activation and enrichment of monocytes and activated DCs may impair PfSPZ VE in relatively malaria-naive individuals but may protect against natural Pf infections in individuals receiving placebo or suboptimal PfSPZ vaccine doses.

To validate these findings, we performed GSEA on baseline whole-blood RNA-Seq transcriptomes from 3 smaller PfSPZ vaccine trials — the VRC312 and VRC314 trials conducted in malaria-naive U.S. adults (7, 9) and the BSPZV1 trial conducted in malaria-experienced Tanzanian adults (17, 18) — limiting to regimens with uniform doses administered by DVI (Supplemental Figure 4A and Supplemental Table 1). Protection was defined as absence of parasitemia for 3–4 weeks after CHMI challenge. Baseline myeloid and innate inflammatory signatures (monocytes, myeloid DCs, low-density neutrophils, “TLR and inflammatory signaling”, “TNF signaling via NF-κB”) were enriched in NP for 1.8 × 10^6^ PfSPZ infants and the VRC trials of malaria-naive adults but, conversely, enriched in P for the BSPZV1 trial of malaria-experienced adults (Supplemental Figure 4, B and C). Notably, heme, erythroid, and IFN signatures, transcriptomic hallmarks of acute malaria (19), were enriched in NP for the trials conducted in malaria-exposed individuals (KSPZV1 and BSPZV1) (Supplemental Figure 4C). The comparison of multiple PfSPZ vaccine trials across different populations demonstrates that, while the extent of cumulative malaria exposure may determine the effect of innate inflammation and myeloid cells on PfSPZ vaccine–induced protection, baseline signatures typical of more recent malaria are associated with reduced efficacy.

### Baseline myeloid and innate inflammatory signatures correlate with postvaccination CSP-specific B cell responses.

**Figure 3:**
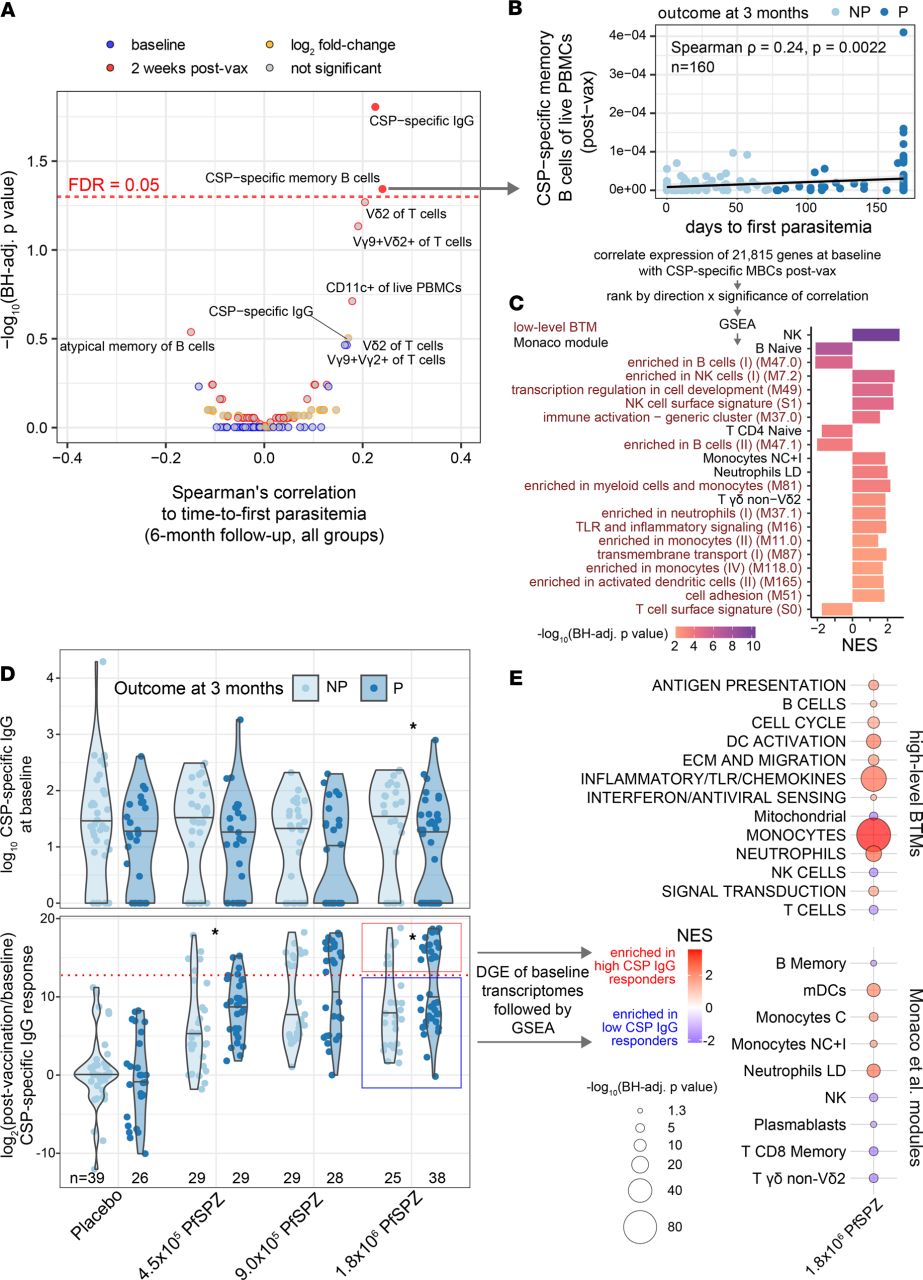
Baseline monocyte and innate inflammatory signatures correlate with postvaccination CSP-specific B cell responses. (**A**) Volcano plot of CSP-specific IgG and flow cytometry features at each time point or calculated as fold-change after vaccination over baseline. (**B**) Correlation between CSP-specific MBCs and time to first parasitemia up to 6 months separated by protected (P) and not protected (NP) outcome at 3 months. (**C**) GSEA using genes ranked by direction and significance of correlation between baseline expression and percentage of CSP-specific of MBCs at 2 weeks after vaccination. (**D**) CSP-specific IgG at baseline and as fold-change (postvaccination/baseline) by treatment and outcome. *P < 0.05 between outcomes within a treatment by Wilcoxon test. Dotted line indicates threshold for high-CSP IgG response. (**E**) GSEA using genes ranked by direction and significance of DGE at baseline between 1.8 × 10^6^ PfSPZ vaccine recipients who subsequently had high or low CSP-specific IgG response after vaccination as defined in **D**. For **C** and **E**, only modules with a Benjamini-Hochberg–adjusted *P* < 0.05 are shown. NES, normalized enrichment score.

To identify molecular signatures predictive of protective immunophenotypes, we first determined correlations between time to first parasitemia with cell type frequencies at prevaccination baseline, at 2 weeks after vaccination, or the fold-difference between the 2 time points using data previously generated for the clinical study (15). Immunophenotypes that most significantly correlated with time to first parasitemia were prevaccination Vδ2^+^ γδ T cells and postvaccination CSP-specific memory B cells (MBCs), consistent with their previously observed protective associations (9, 12) (Figure 3A). Given this significant, albeit weak, correlation, we used postvaccination CSP-specific MBCs as a surrogate for a partially protective response (Figure 3B). Baseline myeloid and innate activation signatures positively correlated with postvaccination CSP-specific MBCs across all PfSPZ groups (Figure 3C). We next assessed CSP-specific IgG reactivity, which also modestly associated with protection (Figure 3A). For 1.8 × 10^6^ PfSPZ, CSP-specific IgG was significantly higher at baseline in NP versus P (Figure 3D), suggesting that preexisting antisporozoite Abs may inhibit high-dose PfSPZ VE. PfSPZ vaccination induced high CSP-specific IgG titers in both NP and P across all doses, suggesting that CSP-specific IgG may not be a reliable mechanistic correlate of protection for this vaccine. When comparing fold change of CSP-specific IgG to account for preexisting Abs, higher vaccine-induced CSP-specific IgG was observed in P versus NP for both 4.5 × 10^5^ and 1.8 × 10^6^ PfSPZ. Taking advantage of the bimodal CSP-specific IgG response to PfSPZ vaccine, we performed DGE followed by GSEA between high and low CSP-specific IgG responders for 1.8 × 10^6^ PfSPZ (Figure 3E and Supplemental Table 2). High CSP-specific IgG responders were enriched for genes related to innate myeloid cell lineages, “inflammatory/TLR/chemokines”, “DC activation”, and “antigen presentation” prevaccination. In contrast, low CSP-specific IgG responders were enriched for lymphocytic signatures at baseline, particularly those of cytotoxic lymphocytes and, to a lesser extent, MBCs and plasmablasts, the latter of which may reflect recent infections that hamper Ab responses to the PfSPZ vaccine. These data suggest that vaccine-induced Pf-specific MBC and Ab responses are enhanced by prevaccination enrichment of antigen presenting cells (APCs) and innate activation.

### Peripheral gene signatures induced by high-dose PfSPZ vaccination predict protection from parasitemia.

**Figure 4:**
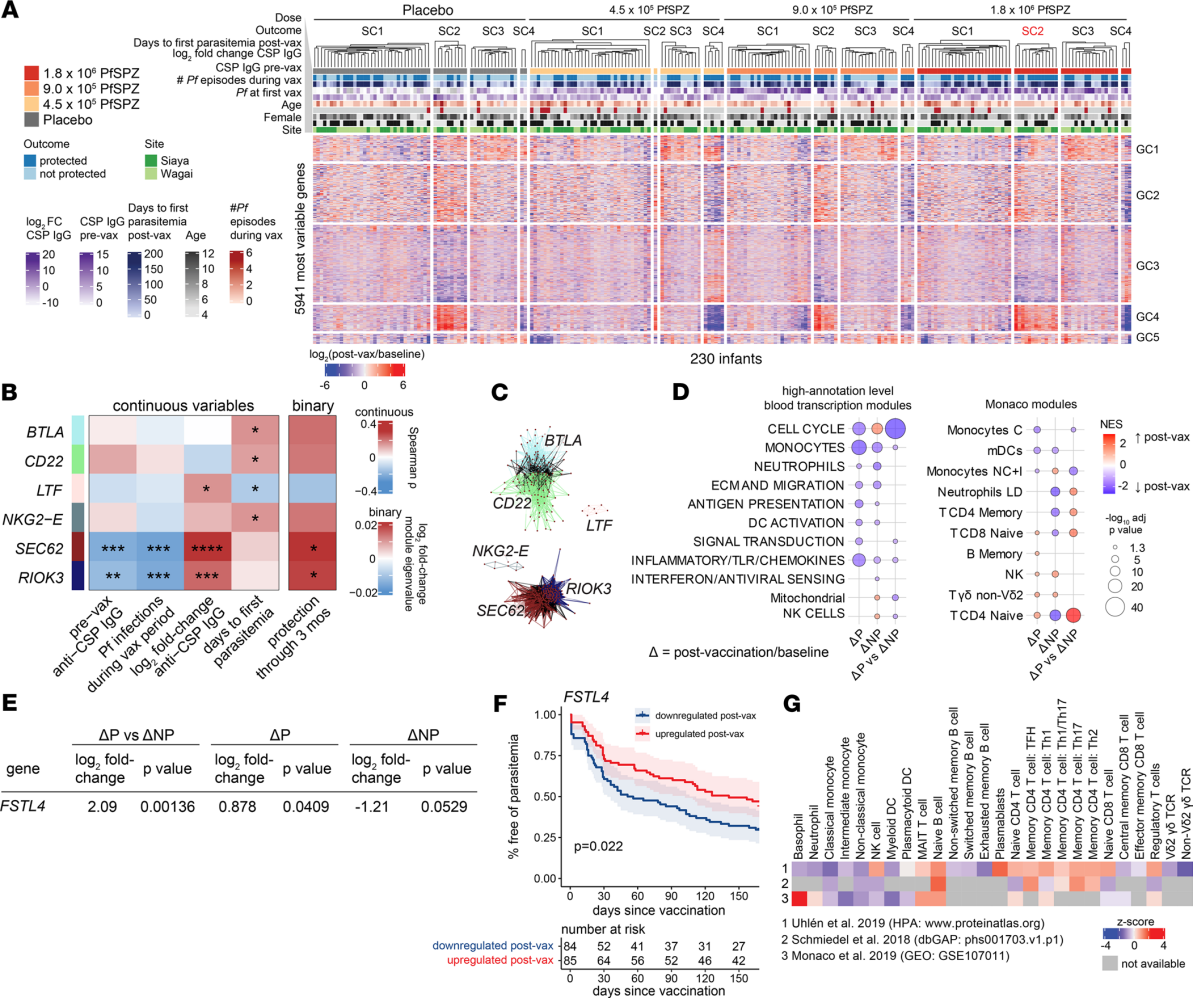
Peripheral gene signatures induced by high-dose PfSPZ vaccination predict protection from parasitemia. (**A**) Unsupervised clustering heatmap of transcriptomic changes for the top 25% most variable genes split by treatment. Ward.D2 and Euclidean distance metric used for clustering samples (SC) and genes (GC). (**B**) Associations of the module eigengenes, obtained by weighted gene correlation network analysis of changes in gene expression (postvaccination/baseline) for 230 infants, with indicated variables determined by Spearman’s correlation or empirical Bayes moderated t test (*P* < 0.05) as appropriate. (**C**) Network graphs of modules in **B** containing nodes (genes) and edges (correlations). (**D**) GSEA of genes ranked by differential expression between postvaccination versus baseline (Δ) within the protected (ΔP) or not protected (ΔNP) groups or between outcomes adjusting for baseline (ΔP versus ΔNP) for 1.8 × 10^6^ PfSPZ infants. (**E**) Genes differentially expressed between ΔP and ΔNP (log_2_ fold-change > 2, *P* < 0.005) in 1.8 × 10^6^ PfSPZ. (**F**) Kaplan-Meier plot of risk of parasitemia for PfSPZ-vaccinated infants with or without upregulation of indicated gene 2 weeks after vaccination. Significance determined by log-rank analysis. (**G**) *FSTL4* expression in human PBMCs across publicly available flow-sorted RNA-Seq data sets.

To determine whether transcriptomic changes during the vaccination period can prospectively identify protected infants, we examined paired differences in blood transcriptomes at 2 weeks after vaccination relative to baseline. Unsupervised clustering of gene expression changes over the vaccination period (Δ gene expression) revealed a sample cluster (SC2) within 1.8 × 10^6^ PfSPZ that was overrepresented by P infants compared with placebo (Figure 4A and Supplemental Figure 5A). Among SC2 infants in 1.8 × 10^6^ PfSPZ, CSP-specific IgG responses at baseline or after vaccination were not significantly different between outcomes and from the other sample clusters (Supplemental Figure 5B). Overrepresentation of P infants within 1.8 × 10^6^ PfSPZ SC2 also could not be explained by differences in parasitemia or study site (Supplemental Figure 5C). The clustering analysis suggests that Δ gene expression could identify favorable responses to high-dose PfSPZ vaccine independently of CSP-specific Abs or confounders of malaria risk.

To assess the relationship between highly correlated Δ gene expression during the vaccination period and outcomes, we constructed data-driven modules for 230 infants with paired transcriptomes using WGCNA and correlated module eigenvalues to parasitemia and CSP-specific IgG. Five modules positively correlated with either protection or days to first parasitemia (Figure 4B). Modules hubbed by *RIOK3* and *SEC62* negatively correlated with prevaccination CSP-specific IgG and Pf infections during the vaccination period while strongly correlating with CSP-specific IgG Δ (Figure 4B and Supplemental Figure 6A). Modules hubbed by *BTLA*-*CD22*, *RIOK3*-*SEC62*, and *NKG2E* formed distinct correlation networks (Figure 4C) and were overrepresented by B cell, heme metabolism, and NK/cytotoxic genes, respectively (Supplemental Figure 6B). Conversely, the *LTF*-hubbed module, which negatively correlated with days to first parasitemia but positively correlated with CSP-specific IgG Δ (Figure 4B), was significantly overrepresented by “inflammatory/TLR/chemokines” genes (Supplemental Figure 6B). The data-driven network analysis suggests that changes in gene signatures related to B cells, cytotoxic lymphocytes, inflammation, and erythrocytes during the vaccination period can affect protection from parasitemia.

For 1.8 × 10^6^ PfSPZ vaccinated infants, paired comparisons of postvaccination versus baseline transcriptomes by DGE within the protected (ΔP) or NP (ΔNP) outcomes adjusted for sex, study site, baseline CSP-specific IgG, and Pf infections during the vaccination period revealed predominantly negative enrichment of innate modules (Figure 4D). Direct comparison between ΔP and ΔNP revealed significant reduction of “cell cycle” and “inflammatory/TLR/chemokines” module expression after vaccination. For cell-type modules, naive CD4^+^ and CD8^+^ T cell, memory CD4^+^ T cell, and low-density neutrophil signatures were significantly induced after vaccination in ΔP relative to ΔNP, whereas monocyte signatures were significantly reduced after vaccination. These differences could reflect trafficking of cell subsets to and from peripheral blood. A single gene *FSTL4* was significantly induced in ΔP versus ΔNP after 1.8 × 10^6^ PfSPZ vaccination using exploratory thresholds (log_2_ fold-change > 2, *P* < 0.005) (Figure 4E and Supplemental Table 3). When applied across all vaccinated groups, *FSTL4* induction was significantly associated with reduced risk of incident parasitemia, even when adjusted for sex, site, baseline CSP-specific IgG, and baseline parasitemia (Figure 4F and Supplemental Table 4). *FSTL4*, predicted to encode a follstatin-like protein with calcium-binding activity, is predominantly expressed in B cells and memory CD4^+^ T cells (Figure 4G) (20–22).

### Integrated multimodal analyses reveal features predictive of PfSPZ-induced protection from parasitemia.

**Figure 5:**
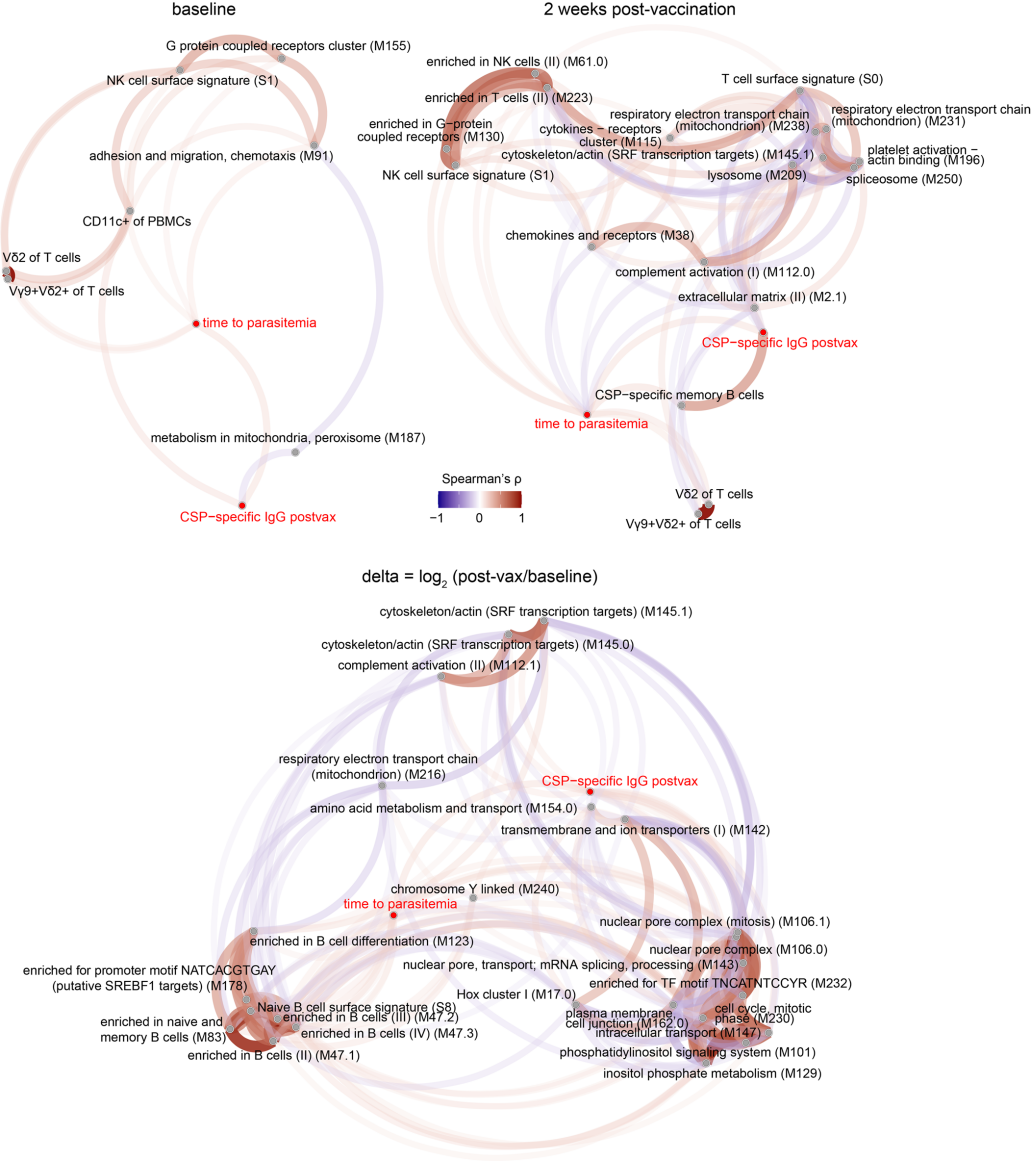
Multimodal correlation analyses reveal features associated with CSP-specific Ab response and protection from parasitemia across all dose groups. Pairwise Spearman’s correlations between baseline, 2 weeks after vaccination, and ∆ features with the outcomes of postvaccination CSP-specific IgG and time to parasitemia at 6 months for all infants with available data (FDR < 5%). Features included module expression scores and flow cytometric data with the addition of plasma cytokines for baseline analysis.

We integrated transcriptomic data with cellular data — including immunophenotyping of rested and PfSPZ-stimulated PBMCs and frequency of CSP-specific B cells — and plasma cytokines to determine significant monotonic relationships with postvaccination CSP-specific IgG and 6-month time-to-parasitemia outcomes across all groups. To reduce features and aid interpretability, gene expression was collapsed to module expression scores using low-level annotation blood transcription modules (BTMs) (23). Significant correlations (FDR < 5%) had weak-to-moderate effect sizes (0.14 < |ρ| < 0.55). Among baseline features, “adhesion and migration, chemotaxis”, Vγ9^+^Vδ2^+^ T cells, “NK cell surface signature”, and “G protein coupled receptors cluster” positively correlated with time to parasitemia, whereas CD11c^+^ PBMCs positively correlated with postvaccination CSP-specific IgG (Figure 5 and Supplemental Table 5). Using postvaccination data, time to parasitemia positively correlated with CSP-specific MBCs and IgG (also shown in Figure 3A), “enriched in G−protein coupled receptors”, Vγ9^+^Vδ2^+^ T cells, and NK and T cell signatures but negatively correlated with “chemokines and receptors”, “extra cellular matrix”, “complement activation”, and “cytokines-receptors cluster”. CSP-specific IgG after vaccination positively correlated with “cytoskeleton/actin” and “platelet activation–actin binding” but negatively correlated with mitochondrion, T cell, and spliceosome signatures. When features were expressed as the Δ, time to parasitemia positively correlated with B cell signatures and “putative SREBF1 targets” but negatively correlated with “chromosome Y-linked” and “Hox cluster I” modules. Postvaccination CSP-specific IgG positively correlated with “intracellular transport” and “complement activation” but negatively correlated with “plasma membrane, cell junction”, “amino acid metabolism and transport”, and “chromosome Y-linked” signatures.

**Figure 6:**
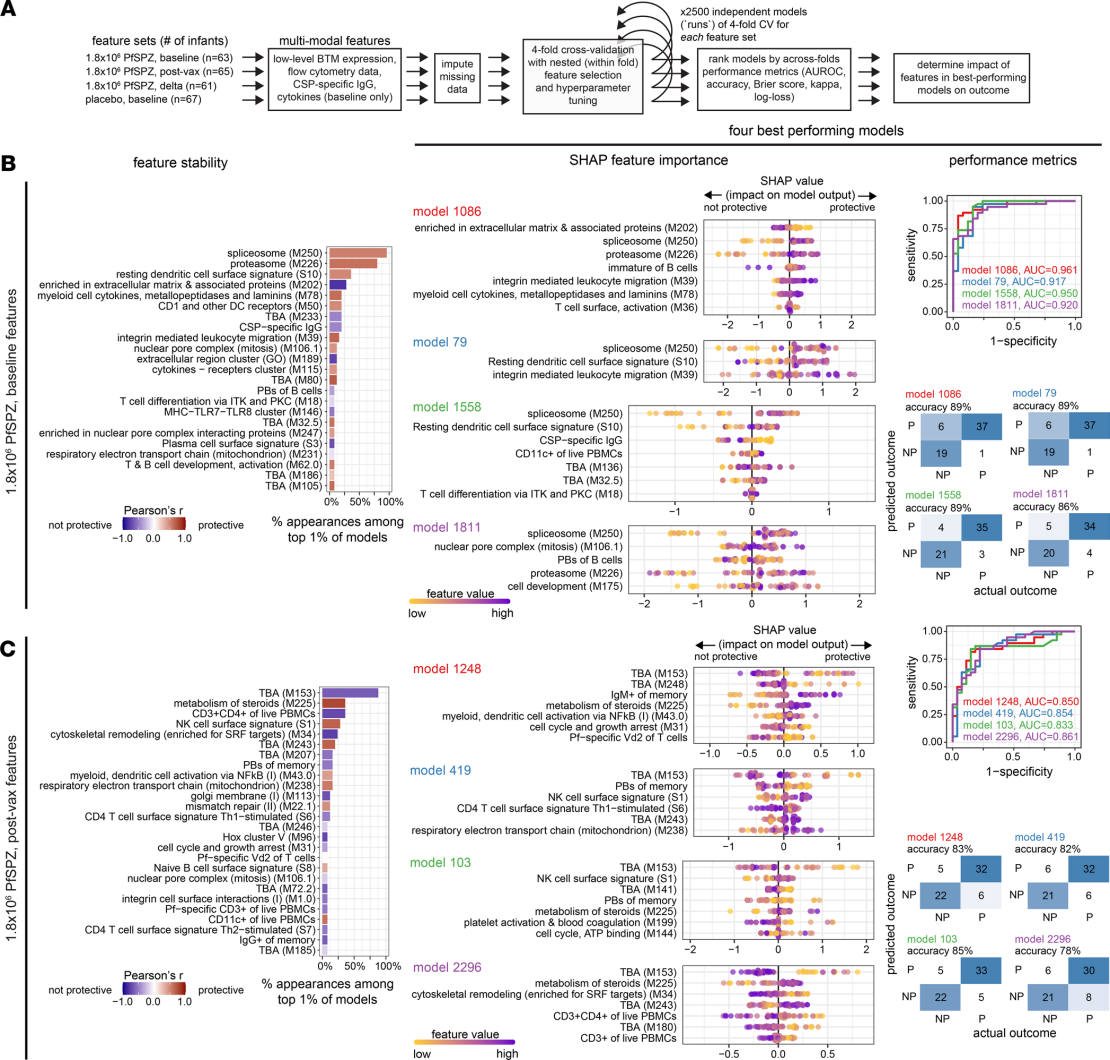
Integrated multimodal machine learning reveal features predictive of PfSPZ-induced protection from parasitemia. (**A**) Overview of machine learning workflow using XGBoost to predict outcome (P versus NP through 3 months) using multimodal models that combined BTM features with flow-cytometric, CSP-specific IgG, and cytokine features. (**B** and **C**) Feature stability plots, SHapley Additive exPlanations (SHAP) plots, and performance metrics are shown for 1.8 × 10^6^ PfSPZ, baseline, (**B**) and 1.8 × 10^6^ PfSPZ, after vaccination (**C**). Feature stability plots show the most common features among the top 1% of 2,500 models evaluated for each feature set. SHAP plots and performance metrics are shown for the top 4 models.

**Figure 7:**
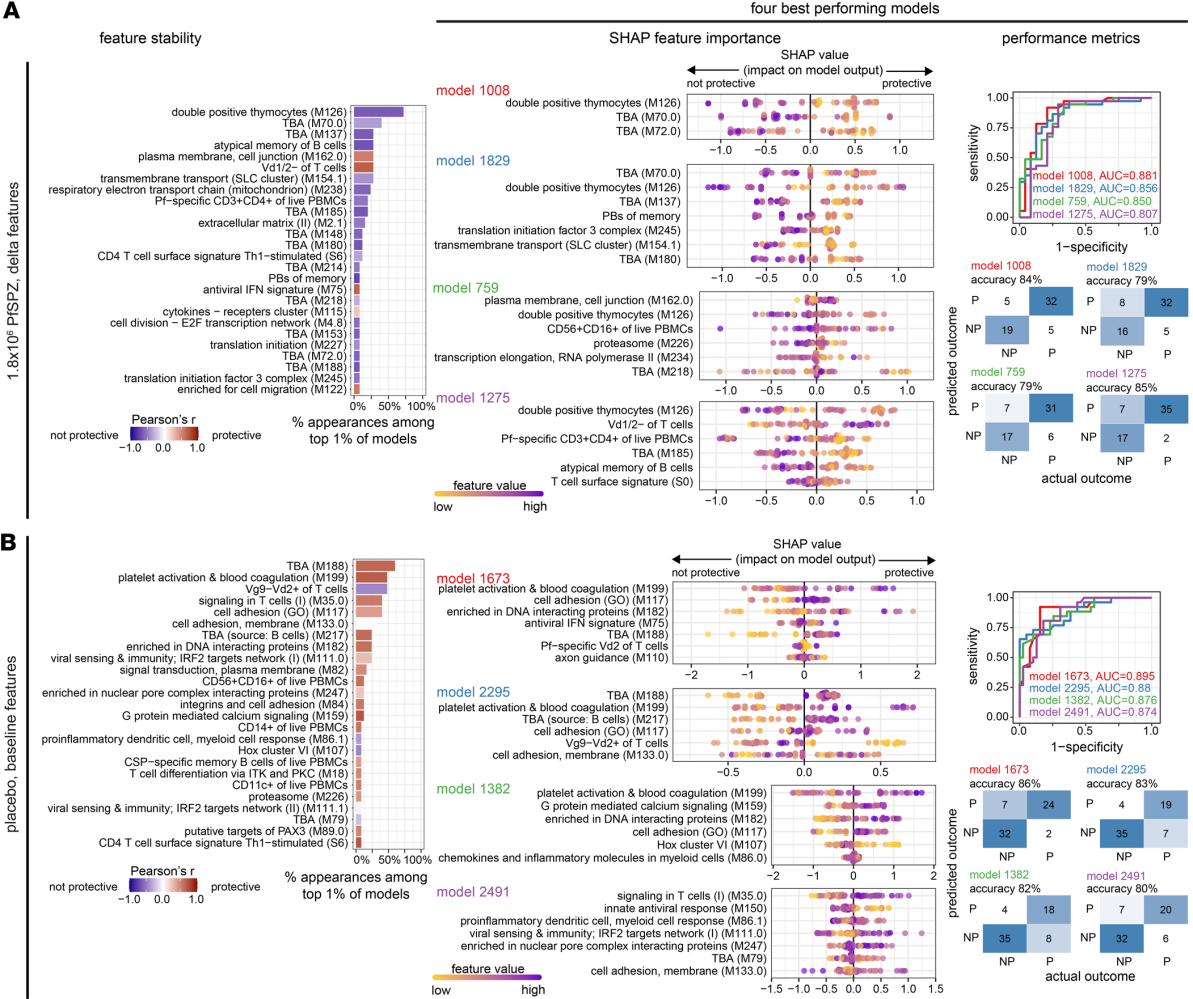
Integrated multimodal machine learning reveals predictive features for ∆ 1.8 × 10^6^ PfSPZ and baseline placebo. (**A** and **B**) Feature stability plots, SHAP plots, and performance metrics are shown for ∆ 1.8 × 10^6^ PfSPZ (**A**) and) baseline placebo (**B**). Refer to Figure 6 for additional details.

We hypothesized that an orthogonal, nonlinear machine learning (ML) approach may identify additional features predictive of outcomes for 1.8 × 10^6^ PfSPZ. Using Extreme Gradient Boosting (XGBoost), we trained and cross-validated multimodal models using either baseline, postvaccination, or Δ data sets that included low-level annotation BTM expression scores, plasma cytokines, flow-cytometric immunophenotypes, and CSP-specific IgG as features (Figure 6A). Overall, the baseline models performed better than the postvaccination and ∆ models (Figure 6, B and C; Figure 7A; and Supplemental Table 6). Among the top performing baseline 1.8 × 10^6^ PfSPZ models, “splicesome”, “proteasome”, and “resting DC surface signature” BTMs appeared most frequently as predictive features and predicted protection (Figure 6B). Notably, the top 4 baseline models included nontranscriptomic features of prevaccination CSP-specific IgG, which predicted nonprotection, and CD11c^+^ PBMCs (primarily monocytes and DCs), which predicted protection (Figure 6B and Supplemental Figure 7A). Both findings are directionally consistent with the significant differences in NP versus P for CSP-specific IgG (Figure 3D) and CD11c^+^ PBMCs (Supplemental Figure 7A). Among the postvaccination models, an uncharacterized module M153 appeared as a feature predictive of nonprotection in nearly all top performing models. The second most frequent postvaccination features were “metabolism of steroids”, which predicted protection, and CD3^+^CD4^+^ PBMCs (CD4^+^ T cells), which predicted nonprotection, with directionality and significance corroborated by Wilcoxon test (Figure 6C and Supplemental Figure 7B). Among the ∆ models, increases in “double positive thymus”, M70.0, and M137 BTMs after vaccination most frequently appeared as nonprotective features. The most frequent nontranscriptomic features were increases in atypical MBCs, which predicted nonprotection, and increases in Vδ1/2^–^ γδ T cells, which predicted protection (Figure 7A and Supplemental Figure 7C).

To further examine the inverse effect of baseline immune activation on protection between 1.8 × 10^6^ PfSPZ and placebo observed by DGE (Figure 2D), ML was applied to placebo baseline features (Figure 7B and Supplemental Figure 7D). The most frequently appearing features among the top performing baseline placebo models were M188, “platelet activation & blood coagulation”, Vγ9^–^Vδ2^+^ T cells, “signaling in T cells (I)”, “cell adhesion”, M217, “enriched in DNA interaction proteins”, and “viral sensing & immunity; IRF2 targets network (I)”. Except for Vγ9^–^Vδ2^+^ T cells, all of these features predicted protection. Among these, “platelet activation & blood coagulation” and “cell adhesion” significantly predicted protection in 3 of the 4 top baseline placebo models, consistent with GSEA results (Supplemental Figure 3B). Features shared across the top 1% of placebo and 1.8 × 10^6^ PfSPZ baseline models were “proteasome”, “enriched in nuclear pore complex interacting proteins”, and “T cell differentiation via ITK and PKC” BTMs, with only “proteasome” appearing in > 50% of top models (1.8 × 10^6^ PfSPZ baseline). The integrated ML analyses suggest that spliceosome, proteasome, and resting DC baseline signatures, along with low baseline CSP-specific IgG, predict high-dose PfSPZ vaccine–induced protection. By comparison, natural protection in placebo is predicted by multiple components of the innate and adaptive immune system, with the most stable protective baseline features identified as “platelet activation & blood coagulation”, “signaling in T cells”, and “cell adhesion”.

### Stimulation of innate immunity can confer protection against liver parasite burden but dampens RAS-induced CD8^+^ T cell responses.

**Figure 8:**
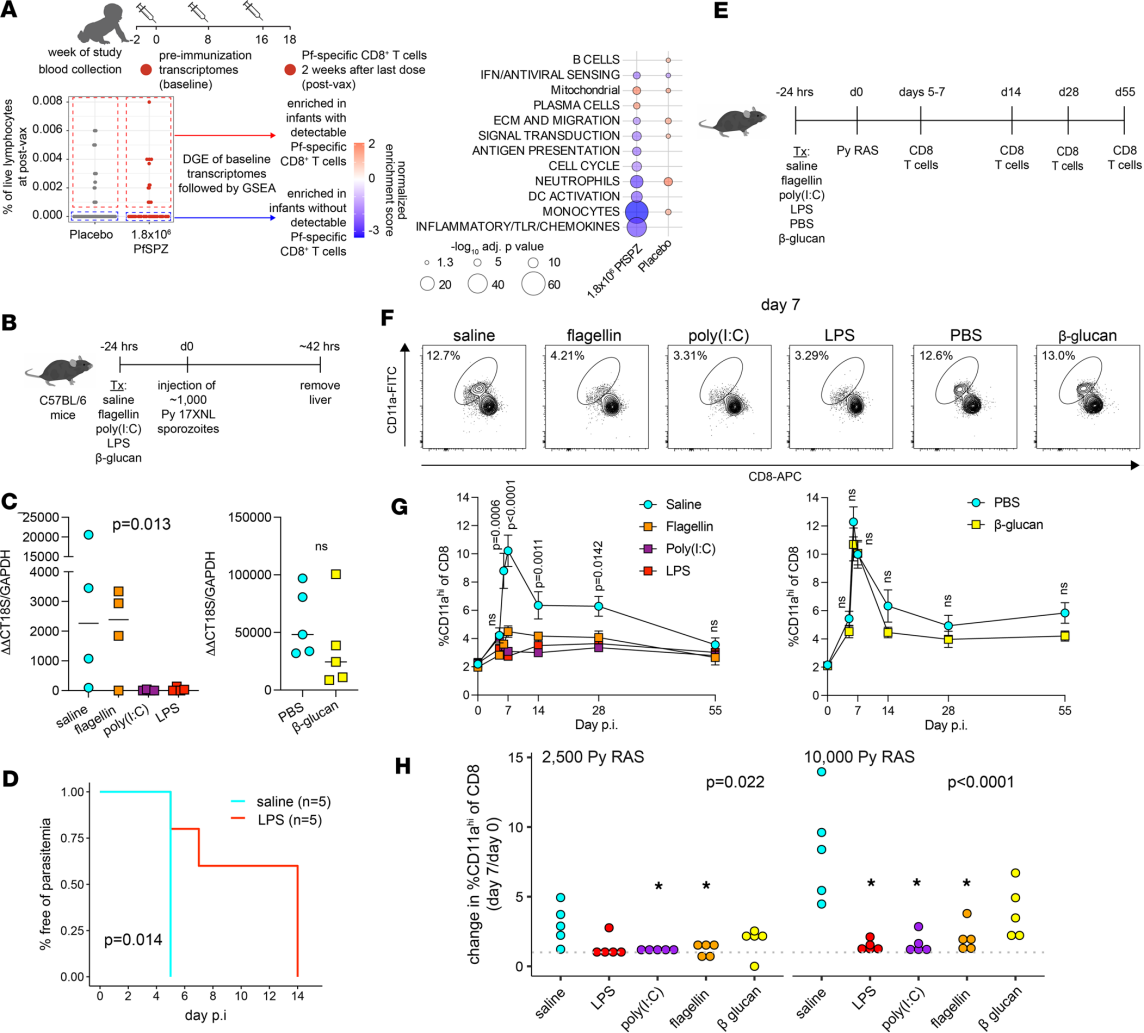
Stimulation of innate immunity reduces liver parasite burden but dampens RAS-induced CD8^+^ T cell responses. (**A**) GSEA using baseline transcriptomes between infants with detectable (red) versus without detectable (blue) PfSPZ-specific CD8^+^ T cell responses. Only BTMs with Benjamini-Hochberg–adjusted *P* < 0.05 are shown. (**B**) Study design for mouse experiments to determine the effect of innate stimuli on *P*. *yoelii* (Py) liver stage infection. (**C**) Liver parasite burden quantification. Each symbol represents a single mouse. Data (median) are representative of 2 independent experiments. Significance determined by Kruskal-Wallis test. (**D**) Kaplan-Meier plot of time to first parasitemia after injection of 1,000 Py 17XNL sporozoites in mice pretreated with LPS or saline for 24 hours. Significance determined by log-rank test. (**E**) C57BL/6 mice were treated with the indicated innate stimuli or control 24 hours before injection of ~1 × 10^4^ Py 17XNL RAS. RAS-induced CD8^+^ T cell responses were enumerated in peripheral blood on the indicated days. (**F**) Representative flow cytometry plots identifying RAS-induced CD8^+^ T cell (CD8^lo^CD11a^hi^). Shown are the percentages of all circulating CD8^+^ T cells that are CD8^lo^CD11a^hi^. (**G**) Percent of circulating CD8^+^ T cells that are CD8^lo^CD11a^hi^ on the indicated day after RAS injection. Data (mean ± SEM) are cumulative results (*n* = 8 mice/treatment) from 2 independent experiments. Significance determined by Kruskal-Wallis test. (**H**) Ratio of circulating CD8^+^ T cells that are CD8^lo^CD11a^hi^ at day 7 after RAS injection over preinfection baseline in 2 experiments independent of those in **G**. Shown are global *P* values for 1-way ANOVA. *P < 0.05 when compared pairwise to saline control by *t* test.

Postvaccination PfSPZ-specific T cell responses were low or undetectable in the KSPZV1 trial (15). Given that innate activation prior to immunization was associated with nonprotection in infants who received 1.8 × 10^6^ PfSPZ vaccine (Figure 2D), we hypothesized that similar innate mechanisms contributed to poor PfSPZ vaccine–induced CD8^+^ T cell responses. Indeed, comparison of baseline transcriptomes revealed increased innate inflammatory (“inflammatory/TLR/chemokines” and “IFN/antiviral sensing”) and myeloid (monocytes, “DC activation”, and neutrophils) signatures in 1.8 × 10^6^ PfSPZ infants without detectable PfSPZ-specific CD8^+^ T cells after vaccination relative to infants with detectable responses (Figure 8A). By contrast, placebo infants with detectable PfSPZ-specific CD8^+^ responses had increased myeloid and B cell signatures at baseline. Notably, baseline IFN signatures were associated with nondetectable responses for both groups. Innate immune activation can inhibit *Plasmodium* liver-stage development and protective adaptive immune responses against the preerythrocytic stage (24–27). We tested the hypothesis that innate stimulation inhibits CD8^+^ T cell priming by reducing liver-stage burden using the *P*. *yoelii* 17XNL (Py) sporozoite infection and RAS immunization models in malaria-naive C57BL/6 mice (Figure 8B). Consistent with a prior study (24), pretreatment with either LPS or the TLR3 agonist poly(I:C) — but neither the TLR5 agonist flagellin nor β-glucan, a dectin-1 agonist that acts via a non-TLR pathway — reduced liver-stage burden after nonirradiated, fully infectious sporozoite injection (Figure 8C). Although none of these pretreatments resulted in complete sterile protection from nonirradiated sporozoites when parasitemia was monitored by PCR, a significant delay in parasitemia was observed for LPS (Figure 8D) but not for β-glucan, flagellin, or poly(I:C) (data not shown). Pretreatment with either flagellin, LPS, or poly(I:C) — but not β-glucan — dampened RAS-induced increases in circulating antigen-experienced CD11a^hi^CD8^lo^ T cells (28) 7–28 days after immunization at both low and high RAS doses (Figure 8, E–H, and Supplemental Figure 8). Taken together, these data suggest that preexisting activation of specific innate signaling pathways can reduce priming of antigen-specific CD8^+^ T cells by RAS.

### Innate immune activation modulates monocyte phagocytic capacity of sporozoites independent of sporozoite-opsonizing antibodies.

**Figure 9:**
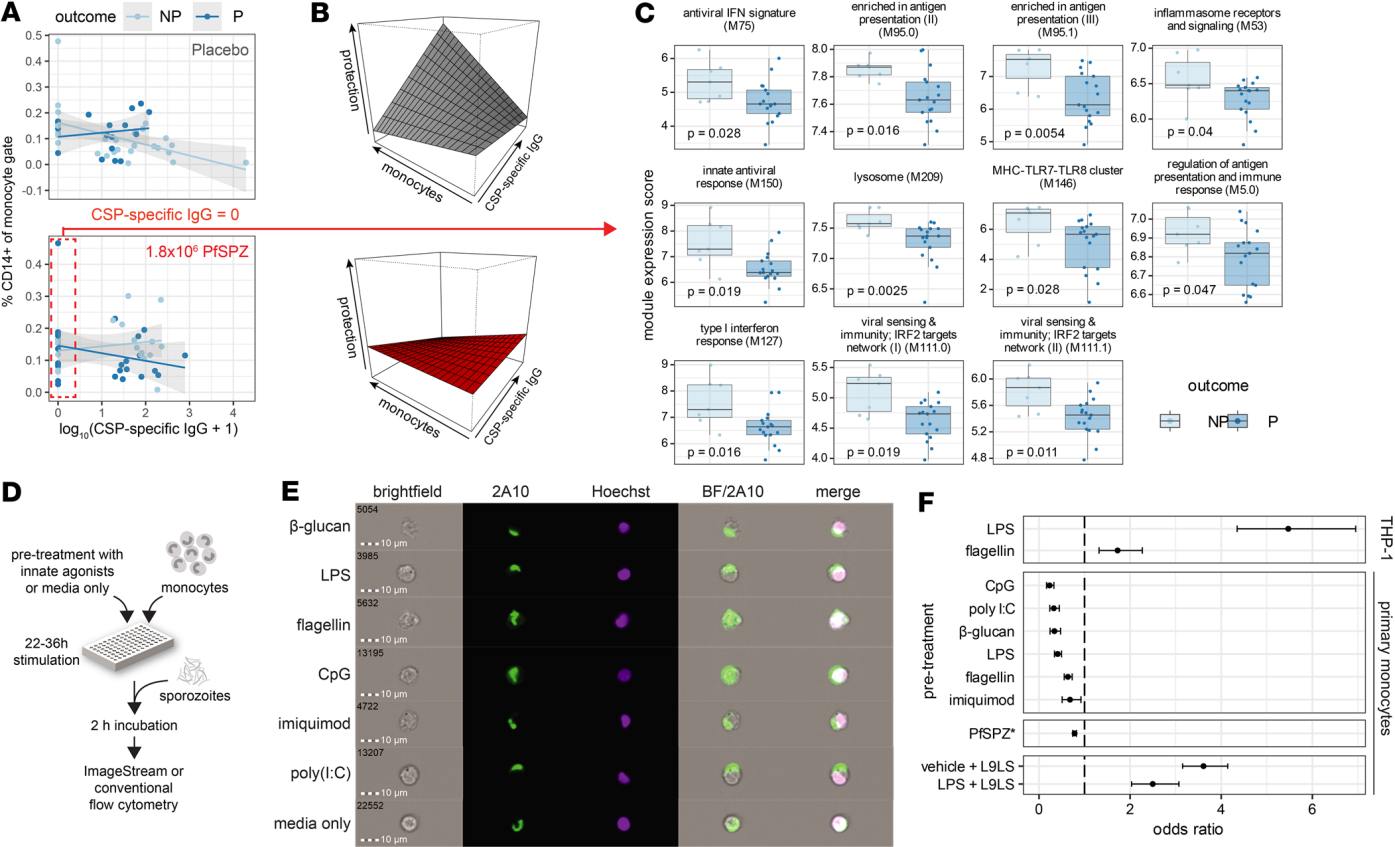
Innate immune activation modulates monocyte phagocytic capacity of sporozoites independent of CSP-specific antibodies. (**A**) Plots of actual values with linear regression fits. (**B**) Perspective plots using fitted values from the logistic regression model in Supplemental Table 7 showing that CSP-specific IgG and CD14^+^ monocytes have differing effects on protection for placebo and 1.8 × 10^6^ PfSPZ groups. (**C**) Baseline expression of innate-related BTMs in nonprotected (NP) and protected (P) infants who received 1.8 × 10^6^ PfSPZ and lacked baseline CSP-specific IgG. (**D**) In vitro sporozoite phagocytosis assay design. (**E**) Representative images of PfSPZ uptake by primary monocytes stained with anti-PfCSP 2A10 Ab after pretreatment with indicated conditions. (**F**) Odds ratios with 95% CIs for number of monocytes containing phagocytosed PfSPZ over total monocytes for indicated treatment versus medium-only control. Opsonization with anti-PfCSP L9LS mAb is shown as a positive control, where reference was isotype control mAb. Significance determined by Fisher’s exact test. Data shown are representative of 2 independent experiments.

Correlation of a baseline gene network related to Fcγ receptor–mediated phagocytosis with protection (Figure 2C) and the association of baseline monocyte signatures and CSP-specific IgG with nonprotection within 1.8 × 10^6^ PfSPZ (Figure 2D and Figure 3D) prompted us to examine these variables in the context of treatment and protection. We observed a significant 3-way interaction between baseline CD14^+^ monocytes, baseline CSP-specific IgG, and treatment (Figure 9, A and B, and Supplemental Table 7) in which increased circulating CD14^+^ monocytes in the presence of CSP-specific IgG predicts protection for placebo but nonprotection for 1.8 × 10^6^ PfSPZ (Supplemental Figure 9A). This treatment-dependent effect of baseline sporozoite-specific IgG and monocytes on protective outcome suggests that Ab-dependent opsonophagocytosis may provide another mechanism that confers short-term protection to natural infection while preventing PfSPZ vaccine–mediated protection. Given the observation that innate immune activation had a similar treatment-dependent effect on outcome (Figure 2, D and E), we asked whether differential innate activation could be seen independently of preexisting CSP-specific IgG. Among 1.8 × 10^6^ PfSPZ infants lacking baseline CSP-specific IgG, innate activation signatures are generally higher in NP versus P, with significance observed for modules related to antiviral responses, IFN, antigen presentation, “activated DCs”, “inflammasome receptors and signaling”, “MHC-TLR7-TLR8 cluster”, and lysosome (Figure 9C and Supplemental Table 8). Conversely, for 1.8 × 10^6^ PfSPZ infants with baseline CSP-specific IgG, expression of the antigen presentation and lysosome modules was increased in P versus NP (Supplemental Table 8). For placebo, no significant differences in module expression were observed between outcomes with or without CSP-specific IgG (Supplemental Table 8).

Preactivation with LPS can augment the opsonin-independent phagocytic efficiency of *Plasmodium*-infected erythrocytes by liver macrophages (29). Based on this finding and our data above, we hypothesized that the reduced liver-stage burden and restricted priming of CD8^+^ T cells after RAS immunization in mice pretreated with LPS and poly(I:C) (Figure 8G) was mediated by enhanced Ab-independent phagocytosis of sporozoites by TLR-activated monocytes. Pretreatment with LPS or flagellin, both which signal via myD88, had contrasting effects on the phagocytosis of Py and PfSPZ depending on monocyte type, with enhancement in the human monocytic THP-1 cell line and inhibition in primary human blood monocytes (Figure 9, D–F, and Supplemental Figure 9, B–D). Conversely, β-glucan and the TLR9 agonist CpG consistently decreased sporozoite phagocytosis by both THP-1 and primary monocytes. Both poly(I:C) and the TLR7 agonist imiquimod decreased phagocytosis of purified sporozoites by human primary monocytes. Nonopsonized sporozoites can induce a regulatory phenotype in monocyte-derived macrophages via upregulation of both activation and regulatory markers (30). To determine whether sporozoite exposure can also modulate primary monocyte function, fresh human monocytes were preexposed to PfSPZ and assessed for activation and nonopsonic phagocytic capacity upon secondary PfSPZ exposure. Preexposure to PfSPZ decreased surface expression of activation markers on peripheral monocytes (Supplemental Figure 10, A and B) and PfSPZ phagocytosis by bystander monocytes (Figure 9F). These data demonstrate that specific innate microbial signals can modulate Ab-independent phagocytosis by monocytes. However, the in vitro evidence favoring inhibition of phagocytic capacity by innate stimuli in primary monocytes suggests that phagocytosis of sporozoites by activated peripheral monocytes is unlikely to be the mechanism by which innate immunity restricts CD8^+^ T cell priming during RAS immunization.

## Discussion

Vaccine-induced protection can be influenced by host intrinsic (e.g., age, genetics) and extrinsic (e.g., preexisting immunity, microbiota) factors (31). Systems analyses of vaccination regimens can elucidate the early immunological processes that drive pathogen-specific adaptive responses and protective efficacy to help identify these factors and inform vaccine design (32, 33). Here, we provide a comprehensive systems analysis of a clinical trial of the PfSPZ vaccine conducted in infants living in a high malaria-transmission setting and identified baseline immune activation and preexisting anti-sporozoite Abs as differentiators of vaccine response.

Several observations from the current analysis are consistent with what is known about sterile immunity to malaria. Baseline enrichment of genes related to NK and γδ T cells in protected versus nonprotected infants for both the placebo and 1.8 × 10^6^ PfSPZ groups may reflect shared cytotoxic gene signatures but is also consistent with their protective roles against liver-stage infection (12, 34). That prevaccination baseline CSP-specific IgG was associated with diminished CSP-specific IgG responses and reduced protective efficacy after high-dose PfSPZ may be consistent with antibody feedback, in which recall responses against immunodominant epitopes are inhibited by preexisting Abs (35). The correlation of vaccine-elicited CSP-specific IgG and MBCs with subsequent protection, although modest, is also consistent with the protective role of neutralizing CSP-specific Abs (36–38). Additionally, despite the lack of Pf-specific cellular responses to PfSPZ vaccine in infants (15), we observed differential enrichment of T cell modules after high-dose PfSPZ vaccination that associated with protection, perhaps reflecting expansion or trafficking of PfSPZ-specific cells.

Our analysis revealed additional insight into innate immunity against Pf infection in malaria-exposed infants. Vγ9Vδ2 T cells can activate and expand in response to sporozoite phosphoantigens, potentially acting as an adjuvant for T cell priming (39). Thus, we previously suggested that the lack of T cell responses in infants may be associated with the relative lack of Vγ9Vδ2 T cells in this age cohort at the time of first immunization (15). However, the current study provides evidence for alternative mechanisms involving innate activation of myeloid cells. Reciprocal enrichment of myeloid and innate inflammatory signatures in protected infants within the placebo group and in nonprotected infants within the 1.8 × 10^6^ PfSPZ group suggests that innate immune activation may confer short-term protection against natural, fully infectious sporozoites but also may prevent the liver-stage infection necessary for PfSPZ vaccine to generate effective CD8^+^ T cell responses and achieve durable protection (10). This was supported by the robust enrichment of baseline innate activation signatures in infants with undetectable Pf-specific CD8^+^ T cell responses relative to those with detectable response.

We also provide in vivo evidence that prestimulation of TLR pathways can restrict priming of antigen-specific CD8^+^ T cells after RAS inoculation by reducing liver-stage burden and delaying time-to-parasitemia after sporozoite infection. We initially hypothesized that TLR agonists increased the capacity of circulating monocytes to phagocytose sporozoites, thereby reducing the liver-stage infection necessary for adequate CD8^+^ T cell priming (6). Although our in vitro experiments using THP-1 cells supported this hypothesis, in primary human monocytes, phagocytosis of sporozoites was reduced after preexposure to different types of innate microbial stimuli, including TLR agonists and even sporozoites themselves. The association between baseline innate activation observed in blood and reduced VE could alternatively be explained by the differentiation of circulating monocytes into CD11c^+^ DCs, which are the APCs responsible for priming CD8^+^ responses (40) and whose baseline “resting” (but not “activated”) signatures were shown to predict protection after high-dose PfSPZ vaccination using ML. LPS could block the conversion of monocytes into DCs (41) or impair cross-presentation (42) to indirectly prevent the priming of liver-stage-specific CD8^+^ T cells.

The innate activation observed in peripheral blood could also reflect systemic activation affecting phagocytic tissue-resident macrophages such as Kupffer cells, which serve as a portal for sporozoites into hepatocytes (43), or hepatocytes themselves. Poly(I:C) can activate Kupffer cells via TLR3 to inhibit sporozoites from progressing to liver-stage (44). Liver-stage infections trigger type I IFN signaling in hepatocytes, which subsequently reduces parasite replication and impairs protective CD8^+^ T cell memory responses against sporozoites (26). Both LPS and poly(I:C) are known to induce type I IFNs via the TRIF/TBK1 pathway (45). This mechanism is supported by our observation that reductions in liver-stage burden and CD8^+^ T cell priming by RAS were observed only for LPS and poly(I:C), which are the only agonists evaluated that signal through TRIF (46). Thus, innate activation may restrict CD8^+^ priming by acting indirectly on hepatocytes to reduce RAS liver-stage progression via type I IFNs induced by TRIF-dependent signaling.

Our findings differ from a transcriptional analysis of Pf-stimulated PBMCs in African children immunized with RTS,S/AS01E that revealed innate, inflammatory gene signatures predicted malaria protection (47). In 1.8 × 10^6^ PfSPZ-vaccinated infants, baseline inflammatory and myeloid signatures negatively correlated with protection but positively correlated with enhanced PfSPZ-induced CSP-specific IgG, consistent with evidence that prevaccination endotypes composed of proinflammatory response genes and derived from innate myeloid cells are predictive of robust Ab responses across multiple vaccines (33). These findings may be explained by mechanistic differences in vaccine-induced protection between RTS,S, which depends on eliciting high-titer CSP-specific IgG (48), and RAS, which relies on generation of Pf-specific CD8^+^ T cells (10). For the PfSPZ vaccine, CSP-specific IgG serves as a weak, nonmechanistic immune correlate of protection, evidenced by lack of efficacy for 9.0 × 10^5^ PfSPZ despite inducing similar CSP-specific IgG titers as 1.8 × 10^6^ PfSPZ.

Comparative enrichment analysis across multiple PfSPZ vaccine trials revealed differences and similarities between this study and the BSPZV1 study of Tanzanian adults. The association of baseline monocyte and TLR/inflammatory signatures with vaccine nonprotection in the malaria-inexperienced cohorts (KSPZV1, VRC312, VRC314) but protection in the more malaria-experienced BSPZV1 cohort suggests that acquired malaria immunity may interact with innate immune activation at baseline to affect PfSPZ VE. In malaria-experienced individuals, preexisting anti-sporozoite Abs may opsonize PfSPZ to favor antigen presentation by peripheral phagocytes rather than by tissue-resident cells after liver-stage infection. However, only in malaria-endemic cohorts (KSPZV1 and BSPZV1) did baseline erythroid and IFN signatures, both induced during acute malaria (19), associate with nonprotection, suggesting that recent malaria infections may be capable of inhibiting PfSPZ VE. In support of this, baseline heme/erythroid-related gene networks positively correlated with preexisting CSP-specific IgG and parasitemia at first immunization. The negative effect of parasitemia on whole-sporozoite immunization is also supported by evidence that Pf parasitemia decreases the efficacy of PfSPZ-CVac, which involves immunization of fully infectious PfSPZ under the cover of chemoprophylaxis (27).

A limitation of a study of natural malaria infection in an endemic population is the potential to misclassify individuals who were not exposed to infectious bites as protected. Given the high entomological inoculation rates (EIR) at the study sites (49), the lack of any malaria exposure over 3 months is unlikely. Differential malaria exposure between individuals would still be a confounder, and this was accounted for in our analysis by adjusting for study site, parasitemia, and preexisting CSP-specific IgG. We did not assess for submicroscopic parasitemia, coinfections, or intestinal microbiota, which may have aided the identification of specific microbial triggers of innate immunity. Given the practical limits for quantity of blood collections in this pediatric field trial, our transcriptomic analysis relied on bulk RNA-Seq of whole blood sampled at only 2 time points. Thus, we could not distinguish differences in expression due to cellular activation versus subset frequency, and a single postvaccination collection reduced sensitivity for detecting Pf-specific cellular responses. Enrichment analyses also relied on available modules that were derived from studies of adults, which may not be wholly reflective of blood signatures in African infants. Future work will be needed to assess the differential kinetics between the blood transcriptomes of vaccine-responders and nonresponders at the single-cell level. We did not investigate whether innate activation affected the phagocytic capacity of macrophages and DCs, which may play more of a role in liver infection than peripheral monocytes. The possible protective role of Ab responses to preerythrocytic antigens other than CSP was also not investigated. Last, since the trial was conducted in a high malaria transmission setting, our findings may not be generalizable to areas with less intense transmission.

In summary, we present evidence supporting a model whereby baseline innate immune activation is associated with short-term protection from natural Pf infection but lack of protective efficacy for the radiation-attenuated whole-sporozoite PfSPZ malaria vaccine. Prevaccination innate immune activation signatures correlated with enhanced vaccine-elicited anti-sporozoite Abs but reduced PfSPZ-specific CD8^+^ T cell responses in infants. Mouse experiments suggest that the loss of protective efficacy by innate immune activation may be via reduced liver-stage infectivity, which restricts priming of antigen-specific CD8^+^ T cell responses by RAS. Taken together, these findings uncouple protective immunity achieved by Abs from cytotoxic responses and suggest that the efficacy of PfSPZ vaccine in malaria-endemic settings might be constrained by opposing antigen presentation pathways. Screening for innate immune activation prior to vaccination could identify those who are the mostly likely to respond to whole-sporozoite malaria vaccine regimens.

## Methods

### Sex as a biological variable.

Sex was included as a covariate in DGE and enrichment analyses. Given that there was no difference in immunogenicity or protective efficacy between male and female children immunized with PfSPZ vaccine (50), only female mice were used for the Py experiments.

### KSPZV1 clinical trial.

Trial details have been described (15, 51). Briefly, the trial was conducted from January 2017 to August 2018 in Siaya County, Kenya, where malaria transmission is highly intense and occurs year-round, peaking during the long (April–July) and short (October–November) rainy seasons. Monthly EIR ranged from 29.9 to 15.7 from October 2018 to September 2019, the closest period for which EIR was available (49). Infants aged 5–12 months were randomized to receive PfSPZ vaccine dosages of 4.5 × 10^5^, 9.0 × 10^5^, or 1.8 × 10^6^ PfSPZ or normal saline placebo administered i.v. 3 times at 8-week intervals. Artemisinin-based combination therapy, primarily artemether-lumefantrine, was administered to all participants 11–19 days prior to the last vaccination to clear parasitemia at the start of malaria surveillance. Parasitemia was determined by active surveillance at scheduled monthly visits and by passive surveillance during symptom-triggered clinic visits, during which a rapid diagnostic test (RDT) or contemporaneous blood smear was performed. Blood smears were prepared but not read in real time unless children had a history of fever, in which case the smear was read immediately. *Plasmodium* infection and parasite densities were determined by 2 certified readers. For discordant results, a third read was carried out. Febrile children were treated for malaria according to RDT or microscopy results. For the current study, the primary outcome was presence of Pf parasitemia (not protected [NP]) or absence of Pf parasitemia (protected [P]) by microscopy through 3 months of surveillance after immunization. We also used a secondary outcome of time (days) to first Pf parasitemia up to 168 days after immunization.

### Clinical sample collection.

Blood samples were collected by venipuncture in PAXgene Blood RNA (BD Diagnostics), serum separator, and K2EDTA (Becton-Dickinson) tubes and were labeled, stored, and shipped in line with Good Clinical Laboratory Practice. Capillary blood drops were used to make thick blood smears and dried blood spots on filter paper.

### RNA processing and RNA-Seq.

For KSPZV1, RNA extraction and sequencing were performed as 2 batches of 4 and two 96-well plates. Individuals were randomized to plates in a treatment-stratified manner with pre- and postvaccination samples from each subject on the same plate. Total RNA was extracted using the PAXgene 96 Blood RNA kit and treated with RNase-Free Dnase Set (Qiagen). RNA quality was assessed on a Fragment Analyzer (Agilent). Average RQN was 8.4. For each sample, 100 ng of total RNA was used for library preparation. Ribosomal and globin mRNA were removed using QIAseq FastSelect rRNA and GlobinRNA removal kits, respectively (Qiagen). RNA was fragmented, converted to cDNA, ligated to index adaptors, and amplified using the KAPA RNA HyperPrep Kit (Roche). Quantification and quality were reassessed. Libraries were pooled with QIAgility (Qiagen). Sequencing of 150 bp paired-end reads was performed on the NovaSeq 6000 Sequencing System v1.0 (Illumina). Illumina sequences were trimmed of contaminating adapters and bases. After assessing sequencing quality using FastQC (v0.11.5, Babraham Bioinformatics), paired-end reads meeting a Phred quality score > 30 were mapped to reference human genome GRCh38 (version 16, Ensembl 99) using STAR RENA-seq aligner (v2.5) and the mapping parameter “Se—outSAMmapqUnique 60”. Assessment of reads distribution was performed using bamutils (ngsutils v0.5.9). Uniquely mapped reads were assigned to hg38 refGene genes using featureCounts (subread v1.5.1) with parameters “-s 2 –p –Q 10”. SeqMonk was used to correct for DNA contamination (Babraham Bioinformatics). Expression of 88 genes encoding lineage markers or relevant to immune responses was validated using nCounter PlexSet (nanoString), with 57 genes (65%) exhibiting Spearman ρ ≥ 0.6 between RNA-Seq and nCounter expression values. For the VRC studies, RNA was extracted from PAXgene tubes using the QIAGEN Rneasy kit with on-column Dnase digestion and rRNA and globin RNA removal. First-strand Illumina-barcoded libraries were generated using the NEBNext Ultra Directional RNA Library Prep Kit for Illumina, using the mRNA capture kit and 12–16 cycles of PCR enrichment. Stranded libraries were sequenced on an Illumina HiSeq 2500 instrument using paired-end 50 bp reads. Data were trimmed for quality using Trimmomatic v0.36 with the following parameters: LEADING:15 TRAILING:15 SLIDINGWINDOW:4:15 MINLEN:37. Trimmed reads were aligned to the hg19 human genome assembly using Bowtie2 v2.2.9. Reads were quantified using HTSeq v0.9.1.

### Immunogenicity analysis.

Immunogenicity studies were done using baseline and 2-week–postvaccination blood samples. Plasma/serum was used for measuring CSP-specific IgG by ELISA as described (15).

### Plasma cytokines.

Plasma cytokines were quantified using the 15-plex human Luminex discovery assay (R&D Systems) at 1:2 dilution and acquired on a Bio-Plex 200 (Bio-Rad).

### Flow cytometry.

PBMCs were used to assess cellular immune responses by multiparameter flow cytometry as previously described (15). Immunophenotyping data for T cells was derived from intracellular cytokine stimulation assays previously performed for the parent clinical trial (15) to evaluate T cell responses elicited by PfSPZ vaccine using previously described methods (9). Briefly, cryopreserved PBMCs were thawed and rested for 8 hours, followed by stimulation for 17 hours with media control or 1.5 × 10^5^ viable, irradiated, aseptic, cryopreserved PfSPZ from a single production lot (PfSPZ-stimulated). Thus, for T cell subsets, media control samples served as an approximation of ex vivo stained PBMCs. Following stimulation, cells were stained and analyzed as described previously (52). Briefly, cells were washed and stained with viability dye, followed by surface stain, cell fixation, and permeabilization with cytofix/cytoperm kit (BD Biosciences) and then by intracellular stain, each for 20 minutes at room temperature (RT). B cell and monocyte surface staining was performed on freshly thawed PBMCs with no rest as previously described (15). See Supplemental Table 9 for a list of Abs used. Upon completion of staining, cells were collected on a FACSymphony flow cytometer (BD Biosciences). Samples were analyzed using FlowJo 10.6.1 (TreeStar). Anomalous “bad” events were separated from “good” events using FlowAI (53). “Good events” were used for all downstream gating. Gating strategies were previously reported (15). The limited cells obtained from infants precluded use of fluorescence-minus-one or isotype controls. Negative and positive gates were determined based on cell populations known to not express the marker of interest.

### Sporozoite preparation.

*Anopheles stephensi* mosquitoes infected with Py were purchased from the Seattle Children’s Research Institute Insectary. Salivary glands were dissected into RPMI medium (Thermo Fisher Scientific). Dissected Py sporozoites were isolated using the Ozaki protocol (54). Purified, cryopreserved, fully infectious Pf and Py sporozoites (PfSPZ and PySPZ) were obtained from Sanaria. Irradiation of the sporozoites was performed using the following parameters: 200 Gy; ~519 cGy/min for 38.5 minutes.

### Sporozoite phagocytosis assays by Imagestream flow cytometry.

The sporozoite phagocytosis assay was modified from a published protocol (55) for use with THP-1 cells and primary monocytes. THP-1 cells were cultured in RPMI medium containing 10% FBS (cRPMI) at 37°C in 5% CO_2_ incubator to a density of 5 × 10^5^ to 1 × 10^6^ cells/mL, whereas primary monocytes were thawed and rested for 2 hours in cRPMI at 37°C in 5% CO_2_. Freshly cultured cells were treated with either LPS from *E. coli* K12 (0.2 μg/mL for primary monocytes; 2 μg/mL for THP-1), poly (I:C) (10 μg/mL), flagellin from *Salmonella typhimurium* (1 μg/mL), imiquimod (2 μg/mL), CpG ODN 2006 (5 μM), or β-glucan (10 μg/mL for primary monocytes; 100 μg/mL for THP-1 cells) for 22–36 hours at 37°C in 5% CO_2_ at 1 × 10^5^ cells/well in 96-well U bottom plates in replicate. Cells were washed with cRPMI and resuspended in fresh medium (15 μL/well). Freshly dissected Py sporozoites, fully infectious PySPZ, or fully infectious PfSPZ were added to pretreated THP-1 or primary monocytes at a sporozoite-to-cell ratio of 1:3 and centrifuged at 500*g* briefly prior to 2-hour incubation at 37°C in 5% CO_2_. Cells were immediately fixed on ice using ice-chilled FoxP3/Transcription Factor Staining Buffer Set (eBioscience) for 10 minites, washed with PBS with 5 mM EDTA (PBS-EDTA), blocked with permeabilization/wash buffer containing 2% BSA, and then washed again with PBS-EDTA. Cells incubated with Py sporozoites were stained with either unconjugated or DyLight-488–conjugated 2F6 mAb specific for PyCSP repeats (56) and were incubated overnight (dissected Py sporozoites) or 45 minutes (PySPZ) at 4°C in the dark. Cells incubated with PfSPZ were stained with DyLight 488–conjugated 2A10 (MR4, BEI resources), a mouse mAb that recognizes the minimal epitope (NANP)_3_ of the PfCSP repeat (57) and incubated for 45 minutes at 4°C with gentle agitation in the dark. Cells were stained with Hoechst 33342 prior to acquisition by Imagestream flow cytometry (Luminex). Cells stained with unconjugated 2F6 mAb were incubated with Alexa Fluor 488–conjugated (AF488-conjugated) goat anti–mouse IgG secondary Ab (Invitrogen; Supplemental Table 9) diluted in blocking buffer at 25°C and washed before nuclear staining. Antibody opsonization of sporozoites was performed using a published protocol with modifications (30). Briefly, PySPZ and PfSPZ were incubated with anti-PyCSP RAM-1 (58) and anti-PfCSP L9LS (38) mAbs (Supplemental Table 9), respectively, at 10 μg/mL for 30 minutes at RT. Sporozoites incubated with isotype control mAbs (InvivoMab, Supplemental Table 9) were used as negative reference controls. Opsonized sporozoites then underwent the same procedure for the sporozoite phagocytosis assay noted above. Analysis of phagocytosis was performed using IDEAS software version 6.0 (Amnis). Briefly, 20,000–30,000 cells were acquired per sample. After exclusion of doublets, debris, and dead cells, the gating strategy in Supplemental Figure 9B was employed. Hoechst^+^ monocytes were examined for internalized sporozoites. The proportion of internal Hoechst^+^CSP^+^/all Hoechst^+^ cells for each treatment condition was compared versus the media-only or isotype control.

### Sporozoite phagocytosis and monocyte activation assays by conventional flow cytometry.

Fresh human peripheral blood, monocytes were negatively selected with the Pan Monocyte Isolation kit (Miltenyi Biotec) from PBMCs isolated from healthy donors using SepMate PBMCs tubes (Stemcell Technologies) per the manufacturers’ instructions. Isolated cells were resuspended in cRPMI, seeded at 1 × 10^5^ cells/well, and incubated at 37°C in 5% CO_2_ incubator for 1 hour. Cells were incubated with CFSE-labeled PfSPZ (1° PfSPZ) at 1:3 sporozoite-to-cell ratio or media control for 2 hours at 37°C in 5% CO_2_. Cells were washed once with cRPMI media and incubated with unlabeled PfSPZ (2° PfSPZ) or media control for another 2 hours. Thus, 4 conditions (no 1° PfSPZ and no 2° PfSPZ [media only]; 1° PfSPZ and no 2° PfSPZ; no 1° PfSPZ and 2° PfSPZ; 1° PfSPZ and 2° PfSPZ) were processed for flow analysis. Cells were washed with cold PBS-FBS, blocked with Fc block at RT, and stained with fixable live/dead dye and fluorochrome-conjugated mAbs HLADR-BV786, CD25-PEcy5, CD80-PE, CD86-BV605, and CD14-PerCPcy5.5 (BioLegend; Supplemental Table 9) at 4°C. Cells were washed with cold PBS-FBS, fixed using ice-chilled FoxP3/Transcription Factor Staining Buffer Set (eBioscience) on ice and were washed with permeabilization/wash buffer prior to staining with AF647-conjugated 2A10 at 4°C. After a final wash in permeabilization/wash buffer, samples were acquired on BD Fortessa flow cytometer and analysis was performed using FlowJo v10 software.

### Mice.

Seven-week-old female C57BL/6 mice (Charles River Labs) were treated i.v. with saline (0.9%; Teknova), flagellin (10 μg; Adipogen), poly(I:C) (200 μg; Tocris), or LPS (10 μg; Sigma) or via i.p. injection with endotoxin-free phosphate-buffered saline (PBS; Corning) or β-glucan (1 mg; MilliporeSigma) 24 hours prior to injection with Py RAS or viable, fully infectious Py sporozoites at 200 μL final volume. CD8^+^ T cell responses were measured following tail vein injection of either 2.5 × 10^3^ or 1 × 10^4^ RAS. Parasite liver burden and prepatent parasitemia were measured following injection of 1 × 10^3^ fully infectious Py sporozoites.

### Liver parasite burden determination in mice.

Mice receiving viable sporozoites were anesthetized ~42 hours after injection using 3.5% isoflurane, 1.5L/min O_2_ prior to euthanasia by cervical dislocation. Livers were removed aseptically and placed in RNAlater (Invitrogen). The left median lobe was dissected, weighed, and bead-mill homogenized in RLT buffer (Qiagen). Liver homogenates were placed on ice prior to RNA extraction (Rneasy Plus Mini Kit, Qiagen) according to the manufacturer’s protocol for liver tissue. RNA purity was assessed followed by cDNA synthesis (ProtoScript II Reverse Transcriptase, New England Biolabs) using the manufacturer’s protocol for random primer mix. cDNA was amplified using Py 18S primers (Supplemental Table 9). Real-time PCR (RT-PCR) was used to quantify relative transcript abundance using a standard curve for the 18S PCR generated with Py stabilite reference cDNA using either PrimeTime 5’ 6-FAM/ZEN/3’ IBFQ probe (Integrated DNA Technologies, Supplemental Table 9) for 18S PCR or SYBR chemistry for the GAPDH PCR (Luna, New England Biolabs) on a QuantStudio 6 Flex RT-PCR System (Thermo Fisher Scientific).

### CD8^+^ T cell quantification.

Peripheral blood from anesthetized mice was collected 24 hours prior to RAS injection and on days 5, 6, 7, 14, 28, and 55 after RAS injection. After RBC lysis, leukocytes were stained with Zombie Aqua (BioLegend), washed, resuspended in FACS buffer containing FC block (anti-CD16/32; clone 2.4G2, BD Biosciences), and stained with anti-mouse mAbs (CD4 PerCP Cy5.5, CD8a BV421, and CD11a FITC; Supplemental Table 9). Cells were washed and fixed prior to a final wash. Labeled cells were acquired on either a BD LSRFortessa X-20 or Attune NxT cytometer and analyzed using FlowJo v.10.7.1.

### Determination of parasitemia in mice.

Blood was sampled by tail snips at regular intervals from days 5–17 after infection. Parasitemia was quantified by flow cytometry by defining parasitized erythrocytes as CD45.2^–^Terr119^+^Dihydroethidium^+^Hoechst^+^ as previously described (59) and by RT-PCR as above but using genomic DNA extracted from blood as template. For flow cytometry, cells were acquired on the Attune NxT cytometer and analyzed using FlowJo v.10.7.1.

### DGE analysis.

For KSPZV1, DGE analysis was performed using edgeR (60) to compare patients who were subsequently P or NP from parasitemia through 3 months after vaccination. After filtering low-expressing genes, remaining genes were normalized by weighted trimmed mean of M-values. Samples with mapped library sizes < 7.5 × 10^6^ counts were excluded. For baseline analysis, filtering and normalization were performed separately for each treatment. After gene-specific dispersion estimation, DGE between outcomes was determined using glmQLFtest and the following, where Pf is parasitemia status at the first vaccination:

 Model matrix: ~Batch + Sex + Site + CSP-specific IgG_baseline_ + Pf + Outcome_3months_

 Contrast: P_3months_ – NP_3months_

Additional DGE analyses were performed on baseline transcriptomes to compare:

High versus low CSP-specific IgG responders:

 Model matrix: ~Batch + Protection + fold-change_CSP_IgG_response

 Contrast: high_CSP_LFC – low_CSP_LFC

Detectable versus not detectable PfSPZ-specific CD8^+^ T cell responses at 2 weeks after vaccination:

 Model matrix: ~Batch + Sex + Site + CSP-specific IgG_baseline_ + Pf + PfSPZ_CD8T_2wkspost-vax_

 Contrast: detectable_2wkspost-vax_ – not_detectable_2wkspost-vax_

Paired analysis of postvaccination samples was performed using *limma* voom (61). The patient was treated as a random effect using duplicateCorrelation. For DGE between postvaccination and baseline time points within an outcome group, the following were used:

 Model matrix: Batch + Sex + Site + CSP-specific IgG_baseline_ + Pf + Outcome_3months__Time point

 Contrasts: P_postvax – P_baseline (ΔP)

 NP_postvax – NP_baseline (ΔNP)

Pf represents the number of Pf infections during the vaccination period, and Outcome_3months__Time point represents the combined parameterization of outcome and time point with baseline as the reference level. To compare the postvaccination effect between outcomes while accounting for baseline, the following contrast was used:

Contrast: ΔP – ΔNP, where Δ denotes postvaccination – baseline within each outcome group.

The VRC312 and VRC314 trials contained multiple vaccine dosing regimens (7, 9). For consistency with the KSPZV1 study, analysis was limited to baseline transcriptomes of patients who received a constant PfSPZ vaccine dose delivered multiple times i.v. The baseline KSPZV1 analysis pipeline was used but with adjustments for sex, dosing regimen, and batch:

 Model matrix: ~Regimen + Sex + Batch_Extraction_ + Batch_Seq_ + Outcome

 Contrast: P – NP

For the BSPZV1 analysis, expression matrices and metadata were obtained from published data sets (GSE196126) and references (14, 18). The baseline KSPZV1 analysis pipeline was used but with adjustment only for dose regimen and baseline CSP-specific Ab titers as sex and batch information was not available:

 Model matrix: ~Regimen + CSP-specific Ab titers_baseline_ + Outcome

 Contrast: P – NP

### Enrichment and regulator analysis.

GSEA (62) was performed with fast GSEA (63) using a custom script that applied the fgseaMultilevel function using a list of all annotated genes ranked by -log_10_(*P* value) × sign(log_2_ fold-change), with significance and fold-change values obtained from the DGE analyses. Minimum gene set size was 20. Gene sets used were low-level (23) or high-level annotation BTMs (64), BloodGen3 modules (65), modules derived from the Monaco data set (22), the MSigDB Hallmark collection, and KEGG pathways. For the Monaco modules, a gene was included within a cell type-specific module if its expression *Z* score (scaled across cell types) was > 1.75.

### WGCNA.

WGCNA (66) was performed using normalized log_2_ counts per million (log_2_CPM) with baseline (*n* = 244) and Δ (log_2_CPM_post-vax_ – log_2_CPM_baseline_, *n* = 230) data. Hub genes were identified for modules with eigengenes significantly associated with protection at 3 months or time-to-parasitemia through 6 months by empirical Bayes moderated *t* test in *limma* and Spearman’s correlation, respectively. To determine biological significance of significant modules, overrepresentation analysis via hypergeometric testing was applied to genes within a network using published gene sets noted above.

### Survival analysis.

Genes meeting the exploratory significance criteria from the ΔDGE analyses for 1.8 × 10^6^ PfSPZ were evaluated as potential vaccine-induced predictors of protection against parasitemia. For each gene, infants from all PfSPZ vaccine groups (*n* = 155) were dichotomized as having expression upregulated or downregulated after vaccination if log_2_(CPM_post-vax_/CPM_baseline_) > 0 or < 0, respectively. The probability of remaining free of parasitemia was estimated using Kaplan-Meier curves. The significance of differences in time-to-incident parasitemia between infants with or without upregulation of each gene was determined by log-rank analysis. A Cox proportional hazards model was used to estimate the risk of parasitemia between groups with age (months), site, parasitemia during the vaccination period, and dose as covariates. The model met the proportional hazards assumptions.

### Correlation analysis of transcriptomic and nontranscriptomic data.

Spearman’s correlations were made for transcriptomic and nontranscriptomic data at baseline, after vaccination, and ∆ (fold-change between postvaccination and baseline status) and are visualized as network plots. For transcriptomic data, gene expression values were transformed as log_2_CPM and were then collapsed into low-annotation level BTMs as module expression scores using the median expression of member genes (346 features). Nontranscriptomic parameters included flow cytometry data, plasma cytokine data (baseline only), postvaccination CSP-specific IgG, and time to parasitemia.

### Class prediction.

Features were used as inputs for classification models were RNA-Seq data (module expression scores), flow cytometric data (immunophenotypes, in vitro stimulation), plasma cytokine concentrations (baseline only), and anti-CSP IgG. The ML algorithms XGBoost (67) support vector machines, and random forest were initially tested to determine which features best predicted outcome for the 1.8 × 10^6^ PfSPZ group. XGBoost was the most consistently accurate algorithm and was selected for further training and cross-validation. Patients missing > two-thirds of features were excluded. For remaining subjects, missing values were imputed using *missMDA*. Four separate analyses were performed using features from baseline 1.8 × 10^6^ PfSPZ, postvaccination 1.8 × 10^6^ PfSPZ, ∆ 1.8 × 10^6^ PfSPZ, and baseline placebo data sets. For each data set, 4-fold cross-validation was performed as 2,500 independent runs with feature selection and hyperparameter tuning nested within each fold. For each run, XGBoost was used for feature selection on ~75% of the samples whereby 3–7 features were randomly selected among the top 10 ranked by importance for further hyperparameter tuning using a random search strategy. Downselected features were then trained using XGBoost with nested cross-validation within each fold to obtain rankings for across-fold performance metrics (accuracy, AUC, Brier score, κ, and log-loss) for each run. Performance of the independent models (2,500/data set) was determined using the average rank across all metrics. Model performance was visualized as ROC curves and confusion matrices. Feature contribution to model prediction output was evaluated using SHapley Additive exPlanations (SHAP) plots.

### Statistics.

Statistical analyses were performed using R software (v4.3.1) or GraphPad Prism (v9.1.0). Specific tests for statistical significance and significance thresholds are described above and in figure and table legends. All *t* tests were 2 tailed.

### Study approval.

Written informed consent was obtained from each infant’s parent/guardian. The clinical protocol (NCT02687373) was approved by IRBs of the Kenya Medical Research Institute, the Centers for Disease Control and Prevention, and the Kenya Pharmacy and Poisons Board. Protocols for secondary use of deidentified human samples and metadata were approved as exempt by the Indiana University IRB (protocol no. 1805696572). Approval for the animal studies was obtained from Indiana University School of Medicine IACUC (protocol no. 19024).

### Data and code availability.

RNA-Seq data and metadata for the KSPZV1, VRC312, and VRC314 analyses are available on dbGaP under the respective accession numbers phs002196.v1.p1, phs002422.v1.p1, and phs002423.v1.p1. Sequence-level data will be made available through a dbGaP controlled access data application. All other data are provided in the Supporting Data Values file and through reproducible code available at https://github.com/TranLab/kspzv1-systems/commit/a843407ca1857508c1f844a3ef968c1801e4753f (commit ID a843407ca1857508c1f844a3ef968c1801e4753f). Additional data visualization apps are available at https://www.kspzv1.malariasystems.org

## Author contributions

TMT and RAS conceived the project. MRL, RBP, LS, EMF, and NWS designed, performed, and analyzed the mouse experiments. JB, RBP, ELG, PAS, DNS, and TMT designed, performed, and analyzed the cellular experiments. KBY, KB, BJF, AY, and WNH sequenced and processed the VRC data sets. MDM, LS, AU, ES, HG, XX, and YL sequenced and processed the KSPZV1 data set. LS, ELG, and ALO performed gene expression validation. LCS, MO, KO, TM, TLR, BKLS, SK, SLH, and RAS designed and conducted the clinical trial. REW, LCS, and TMT analyzed the clinical trials data. TMT, LS, XL, PH, and AU performed the integrated analyses, machine learning, and data visualizations. TMT, LS, JB, MRL, RBP, PH, NWS, LCS, MO, and RAS cowrote the manuscript. The first coauthor order was assigned by time contributed to the project.

## Supplementary Material

Supplemental data

Supplemental table 1

Supplemental table 2

Supplemental table 3

Supplemental table 4

Supplemental table 5

Supplemental table 6

Supplemental table 7

Supplemental table 8

Supplemental table 9

Supporting data values

## Version 1 04/30/2024

In-Press Preview

## Version 2 06/10/2024

Electronic publication
