# Supplementary material for: Innate immune activation restricts priming and protective efficacy of the radiation-attenuated PfSPZ malaria vaccine
Source: JCI Insight. 2024 Apr 30;9(11):e167408. doi: 10.1172/jci.insight.167408 (PMC11382880; doi:10.1172/jci.insight.167408)
Supplement: Supplemental data [file jciinsight-9-167408-s109.pdf]

Supplemental material

Title:

Innate immune activation restricts priming and protective efficacy of the radiation-attenuated PfSPZ malaria vaccine

Authors:

Leetah Senkpeil*[1,2], Jyoti Bhardwaj*[1], Morgan R. Little*[3], Prasida Holla[3], Aditi Upadhye[1], Elizabeth M. Fusco[2], Phillip A. Swanson II[4], Ryan E. Wiegand[5], Michael D. Macklin[1], Kevin Bi[6], Barbara J. Flynn[4], Ayako Yamamoto[4], Erik L. Gaskin[1], D. Noah Sather[7], Adrian L. Oblak[8], Edward Simpson[9], Hongyu Gao[9], W. Nicholas Haining[10], Kathleen B. Yates[6], Xiaowen Liu[11], Tooba Murshedkar[13], Thomas L. Richie[13], B. Kim Lee Sim[13], Kephas Otieno[12], Simon Kariuki[12], Xiaoling Xuei[9], Yunlong Liu[9], Rafael Polidoro[3], Stephen L. Hoffman[13], Martina Oneko[12], Laura C. Steinhardt[5], Nathan W. Schmidt[2,3], Robert A. Seder[4], Tuan M. Tran[#1,2,3]

*Shared first-author.

Affiliations:

[1]Division of Infectious Diseases, Department of Medicine, Indiana University School of Medicine, Indianapolis, Indiana, USA
[2]Department of Microbiology and Immunology, Indiana University School of Medicine, Indianapolis, Indiana, USA
[3]Ryan White Center for Pediatric Infectious Diseases and Global Health, Department of Pediatrics, Indiana University School of Medicine, Indianapolis, Indiana, USA
[4]Cellular Immunology Section, Vaccine Research Center, NIAID, NIH, Bethesda, Maryland, USA
[5]Malaria Branch, Division of Parasitic Diseases and Malaria, Center for Global Health, Centers for Disease Control and Prevention, Atlanta, Georgia, USA
[6]Broad Institute of MIT and Harvard, Cambridge, MA, USA
[7]Center for Global Infectious Disease Research, Seattle Children's Research Institute, Seattle, WA, USA
[8]Stark Neurosciences Research Institute, Indiana University School of Medicine, Indianapolis, Indiana, USA
[9]Center for Medical Genomics, Indiana University School of Medicine, Indianapolis, Indiana, USA
[10]Department of Pediatric Oncology, Dana-Farber Cancer Institute, Boston, MA, USA
[11]Deming Department of Medicine, Tulane University School of Medicine, New Orleans, LA, USA
[12]Kenya Medical Research Institute, Centre for Global Health Research, Kisumu, Kenya
[13]Sanaria, Rockville, Maryland, USA

A

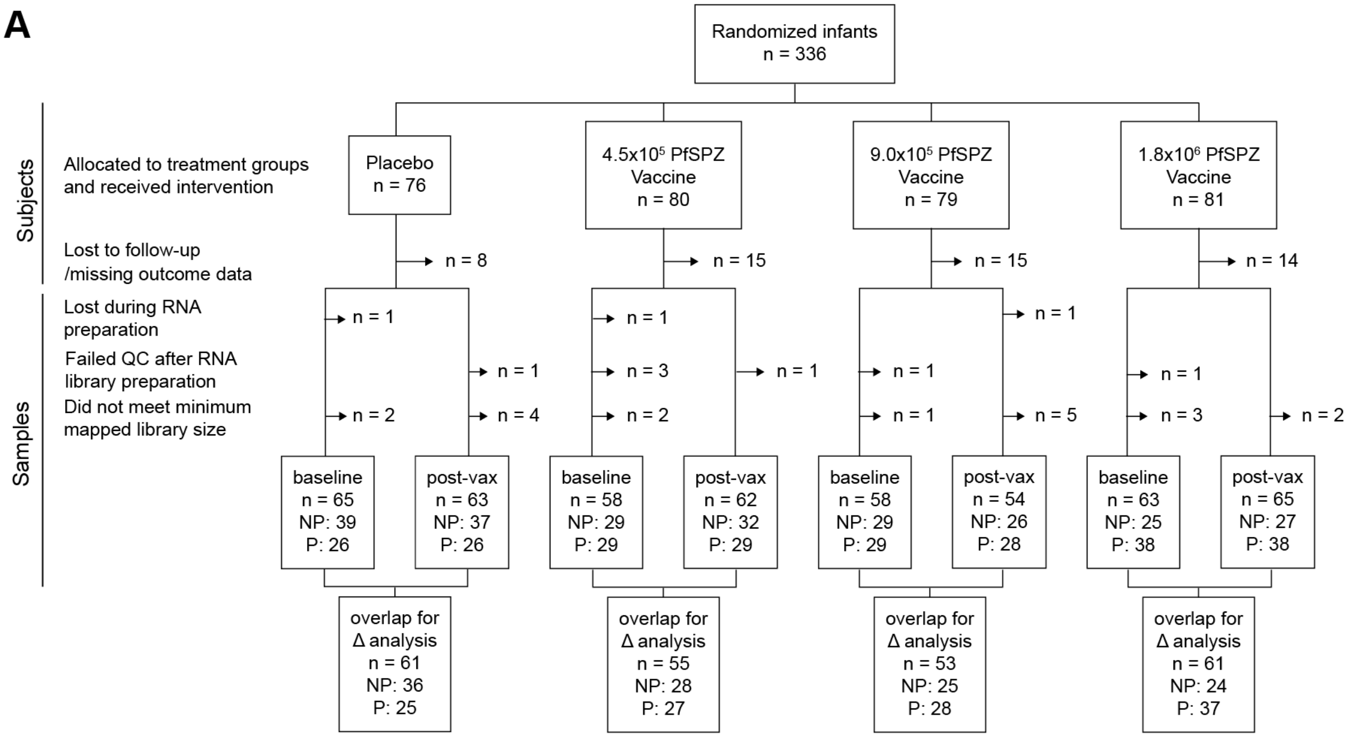

B

	N	not protected (NP)	protected (P)	significance
placebo		(N=40)	(N=26)	
Site : Wagai	66	17 (42.50%)	20 (76.92%)	P=0.012[1]
Sex : M	66	22 (55.00%)	13 (50.00%)	P=0.884[1]
Age	66	6.42 **9.00** 10.00	6.92 **8.50** 11.00	P=0.806[2]
Hemoglobin	66	9.30 **10.00** 10.80	9.88 **10.65** 11.32	P=0.066[2]
Platelets	66	278.08 **394.50** 461.75	333.17 **412.50** 516.50	P=0.256[2]
Weighted_z	66	-0.74 **-0.18** 0.32	-0.57 **0.29** 0.93	P=0.126[2]

	N	not protected (NP)	protected (P)	significance
4.5x10⁵ PfSPZ		(N=30)	(N=29)	
Site : Wagai	59	12 (40.00%)	13 (44.83%)	P=0.911[1]
Sex : M	59	18 (60.00%)	15 (51.72%)	P=0.706[1]
Age	59	7.00 **8.00** 11.00	6.00 **8.00** 11.00	P=0.921[2]
Hemoglobin	59	9.97 **10.85** 11.40	10.30 **10.80** 11.33	P=0.867[2]
Platelets	59	297.08 **376.50** 433.00	347.00 **443.00** 557.33	P=0.058[2]
Weighted_z	59	-1.14 **-0.24** 0.46	-1.14 **-0.06** 0.57	P=0.891[2]

	N	not protected (NP)	protected (P)	significance
9.0x10⁵ PfSPZ		(N=33)	(N=31)	
Site : Wagai	64	17 (51.52%)	12 (38.71%)	P=0.437[1]
Sex : M	64	14 (42.42%)	14 (45.16%)	P=1.000[1]
Age	64	7.00 **8.00** 10.33	6.00 **7.00** 9.00	P=0.166[2]
Hemoglobin	64	8.97 **9.70** 10.87	10.12 **10.60** 11.27	P=0.050[2]
Platelets	64	260.33 **373.00** 486.67	290.00 **444.00** 556.83	P=0.097[2]
Weighted_z	64	-1.07 **-0.31** 0.39	-0.99 **0.05** 0.31	P=0.904[2]

C

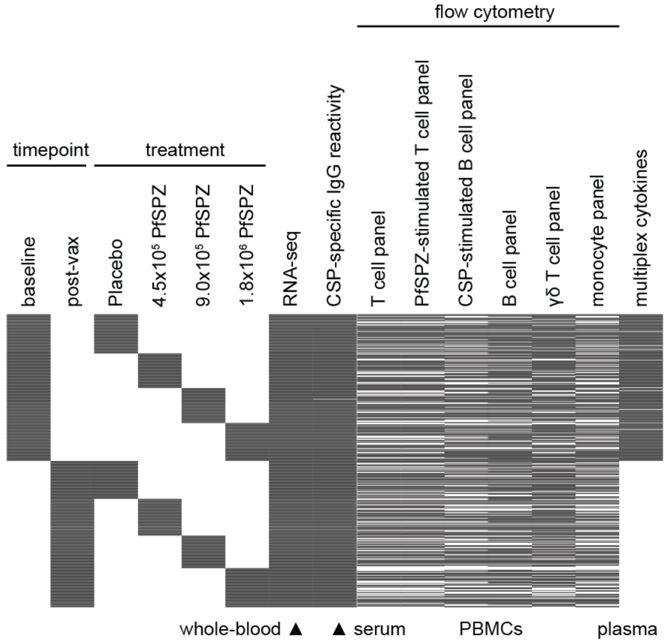

	N	not protected (NP)	protected (P)	significance
1.8x10⁶ PfSPZ		(N=28)	(N=39)	
Site : Wagai	67	12 (42.86%)	21 (53.85%)	P=0.522[1]
Sex : M	67	18 (64.29%)	27 (69.23%)	P=0.872[1]
Age	67	6.00 **8.00** 9.58	6.33 **9.00** 10.00	P=0.239[2]
Hemoglobin	67	9.60 **10.30** 11.22	9.22 **10.40** 11.17	P=0.819[2]
Platelets	67	342.33 **440.50** 555.17	322.83 **432.00** 557.33	P=0.794[2]
Weighted_z	67	-0.75 **-0.06** 0.57	-0.79 **-0.16** 0.50	P=0.576[2]

Figure S1. Participants and samples used in study.

Related to Figure 1. **(A)** Flow diagram of subjects and samples used for transcriptomic analyses. **(B)** Characteristics study participants by outcome and treatment. [1]Two-proportions Z-test and [2]Wilcox test used to determine statistical significance for categorical and continuous variables, respectively. **(C)** Samples used for each assay by time point and treatment.

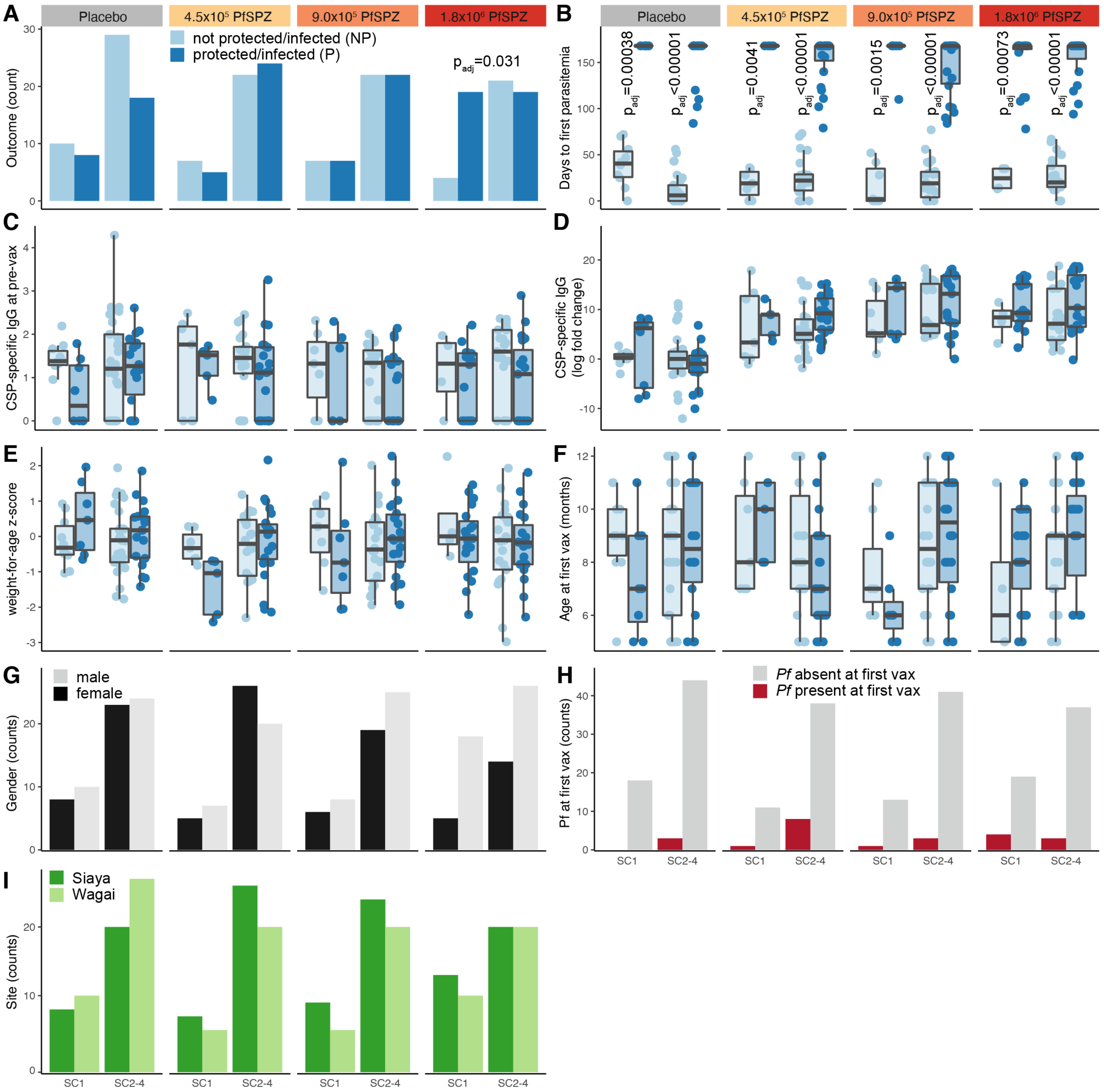

Figure S2. Comparisons within pre-immunization sample cluster 1 (SC1) and SC2-4 by dose group.

Related to Figure 1. **(A-I)** Comparisons of indicated variables within sample cluster 1 (SC1) and SC2-4 by dose group. Outcomes are not protected/infected (NP; dark blue) or protected/not infected (P; light blue) during 3 months of surveillance post-immunization (see Figure 1A). Sample clusters were based on hierarchical clustering of whole-blood transcriptomic profiles at pre-immunization baseline (see Figure 1B). Wilcoxon and Fisher's exact tests used to determine statistical significance for continuous and categorical variables, respectively. For A, Fisher's exact test was performed on 2x2 contingency table (outcome vs SC1 or SC2-4 membership) within each dose, and p values adjusted for four independent comparisons. Shown are Benjamini-Hochberg-adjusted p values (p_{adj}) for comparisons where $p_{adj} < 0.05$.

Table S1. Differential gene expression analysis between protected and non-protected infants for baseline samples by clinical trial and treatment group.

Provided as a separate spreadsheet.

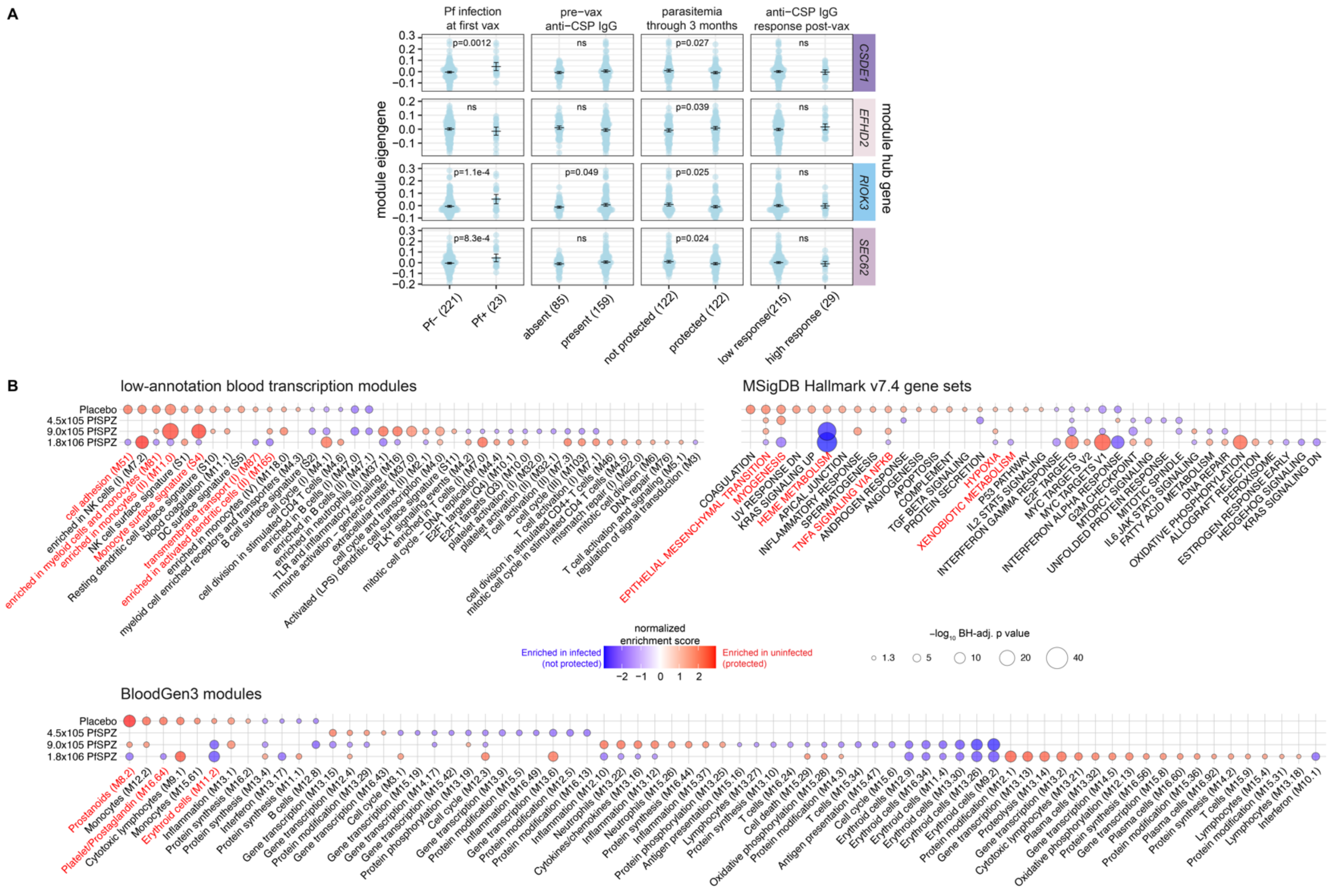

Figure S3. Weighted gene correlation network and gene set enrichment analysis of pre-immunization whole-blood transcriptomes.

Related to Figure 2. **(A)** Beeswarm plots comparing module eigengenes at the pre-immunization baseline for the modules and traits shown Figure 2A for all 244 infants. Group sample sizes are indicated in parentheses. Error bars represent the 95% confidence intervals around the mean. Significance of differences between eigengenes was determined using the empirical Bayes moderated t-statistics test in the limma package with adjustment for study site and dose group. ns = not significant. **(B)** For each dose group, gene set enrichment analysis was performed between infants who were protected (uninfected) through 3 months of follow up versus those who were not protected (infected) using low-annotation level blood transcription modules, MSigDB Hallmark gene sets, and BloodGen3 modules. Modules are sorted from left to right by decreasing normalized enrichment score and treatment. Modules enriched in “protected” for Placebo but enriched in “not protected” for 1.8x10^{6} PfSPZ are highlighted in red text. Only modules with a minimum gene set size of 20 and a Benjamini-Hochberg-adjusted p value (false discovery rate) <0.05 are shown.

A

Study	Country	Malaria Exposure	Group	Regimen	NP	P
KPSZV1	Kenya	malaria-exposed infants	high dose	1,800,000 PfSPZ x 3	24	37
VRC 312	US	malaria-naive adults	3a	30,000 PfSPZ x 6	3	0
			3b	30,000 PfSPZ x 4	8	1
			4a	135,000 PfSPZ x 5	0	2
			4b	135,000 PfSPZ x 5	0	4
			4c	135,000 PfSPZ x 4	3	6
			All		14	13
VRC 314	US	malaria-naive adults	1	270,000 PfSPZ x 3	1	3
			4	270,000 PfSPZ x 4	0	5
			5	270,000 PfSPZ x 4	5	6
			6	900,000 PfSPZ x 3	5	9
			All		11	23
BSPZV1	Tanzania	malaria-exposed adults	2	135,000 PfSPZ x 5	7	4
			3	270,000 PfSPZ x 5	7	4
			All		14	8

B

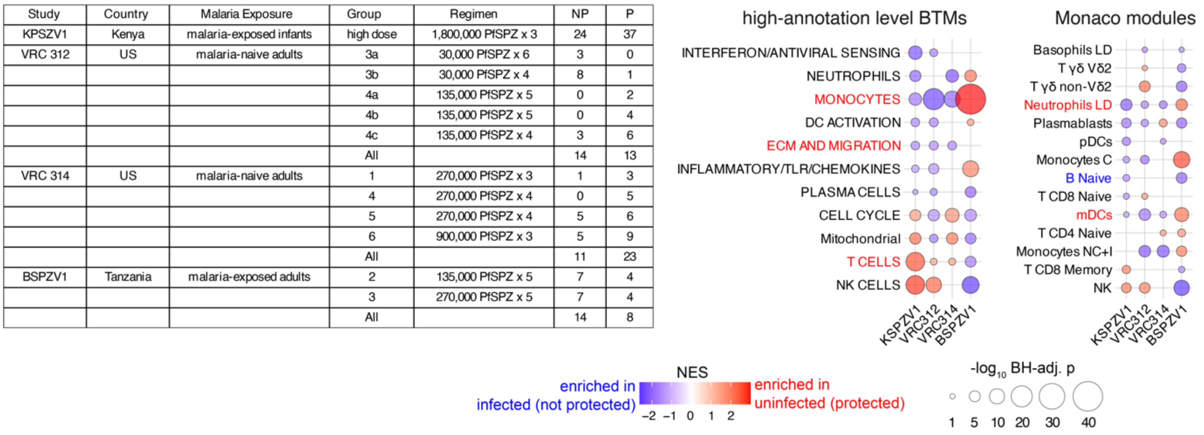

C

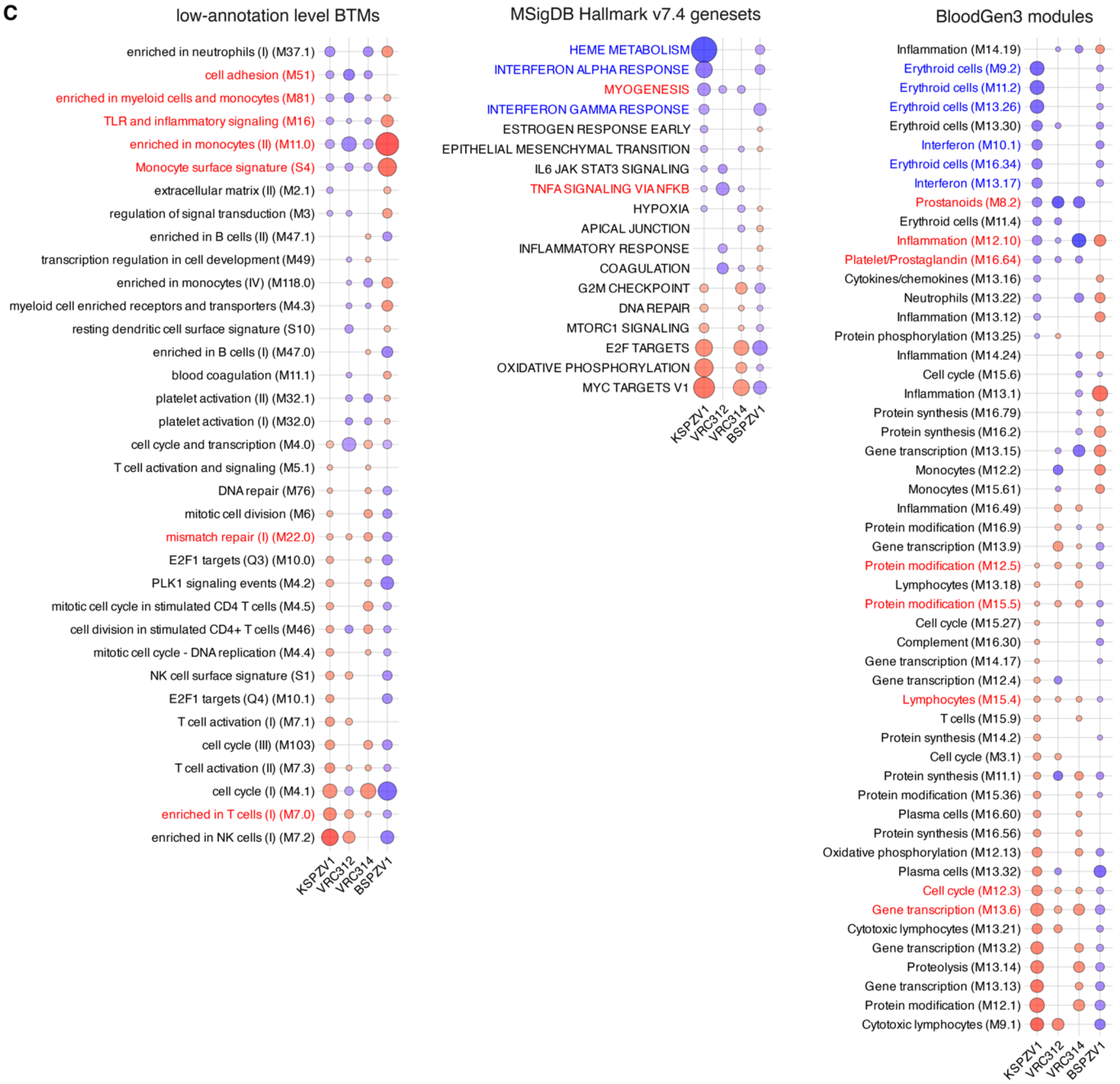

Figure S4. Gene set enrichment analysis of pre-vaccination whole-blood transcriptomes across four PfSPZ Vaccine trials. Related to Figure 2. **(A)** Numbers of protected and not protected subjects by dose regimen and trial with samples available for analysis. For VRC trials, only groups in which a consistent PfSPZ dose was delivered by DVI was included for differential gene expression (DGE) analysis. Gene set enrichment analysis of baseline transcriptomes between protected (P) and not protected (NP) individuals using **(B)** higher and **(C)** lower annotation level modules. DGE analyses included adjustments for batch, dose regimen, gender, and baseline CSP-specific Abs where applicable. BH = Benjamini-Hochberg. Only modules with a minimum gene set size of 20 and -log$_{10}$(BH-adjusted p value) > 1 (equivalent to FDR<10%) in ≥2 studies are shown. Red text: similar across studies of infants, who are relatively malaria-naïve, or malaria-naïve adults. Blue text: similar across studies of individuals who are naturally exposed to malaria.

Table S2. Differential gene analysis of baseline transcriptomes between high- and low-CSP IgG responders (see Figure 3D).

Provided as a separate spreadsheet.

Table S3. Differential gene expression analysis between protected and non-protected infants post-vaccination with adjustment for pre-vaccination baseline for 1.8x10^6 PfSPZ.

Provided as a separate spreadsheet.

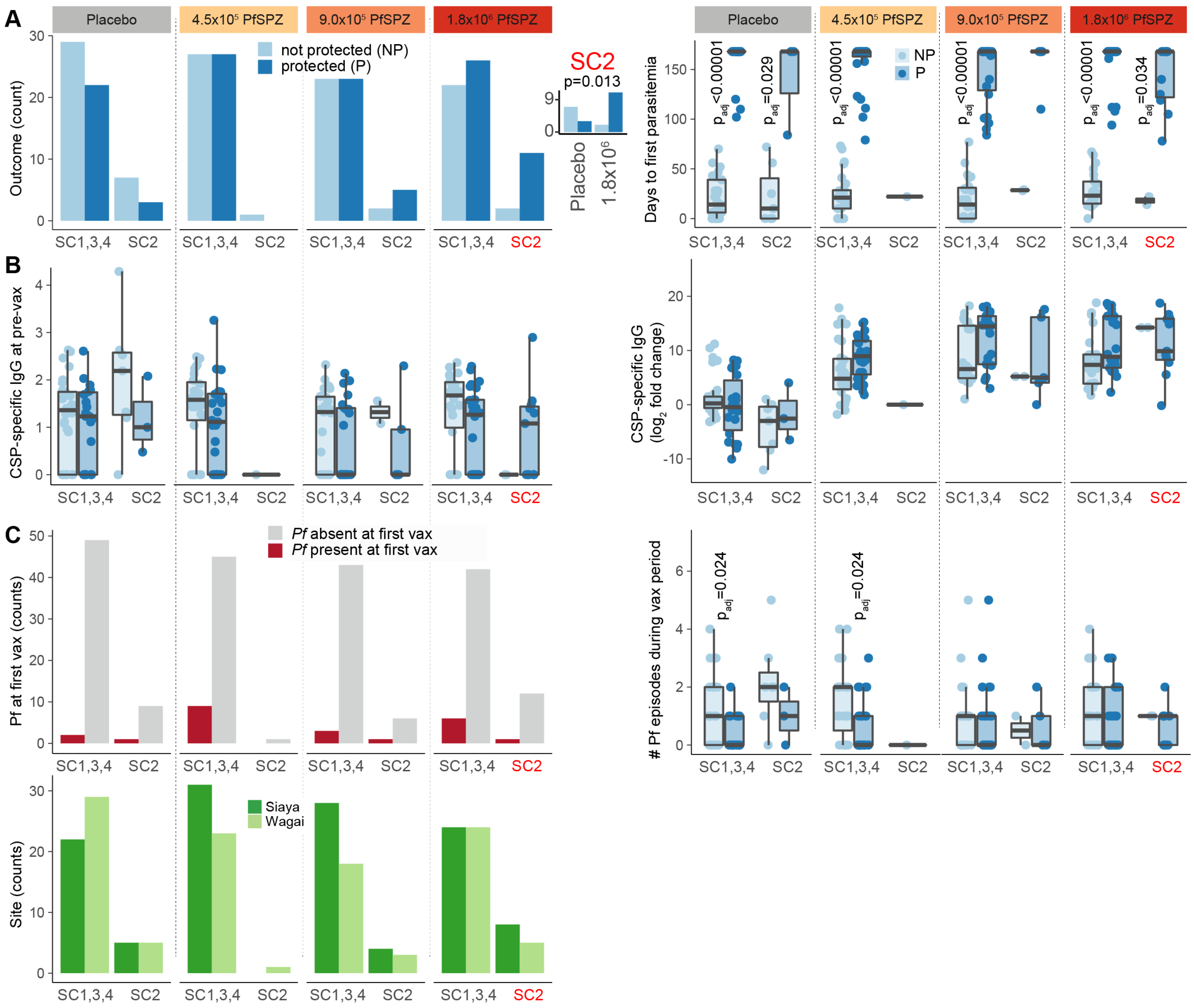

Figure S5. Cluster analyses of changes in post-immunization whole-blood transcriptomes over pre-immunization baseline.

Related to Figure 4. **(A)** Comparisons of indicated variables within delta sample cluster 2 (SC2) and SC1,3,4 by dose group for protection and days to first parasitemia; **(B)** CSP-specific IgG response at baseline and post-vaccination; and **(C)** Pf infections and study site. Outcomes are not protected/infected (NP; dark blue) or protected/not infected (P; light blue) during 3 months of surveillance post-vaccination (see Figure 4A). Sample clusters were based on hierarchical clustering of changes in whole-blood transcriptomes over the vaccination period (post-immunization/pre-immunization baseline = Δ). Wilcoxon test and Fisher's exact used to determine statistical significance for continuous and categorical variables, respectively. Shown are B-H adjusted p values.

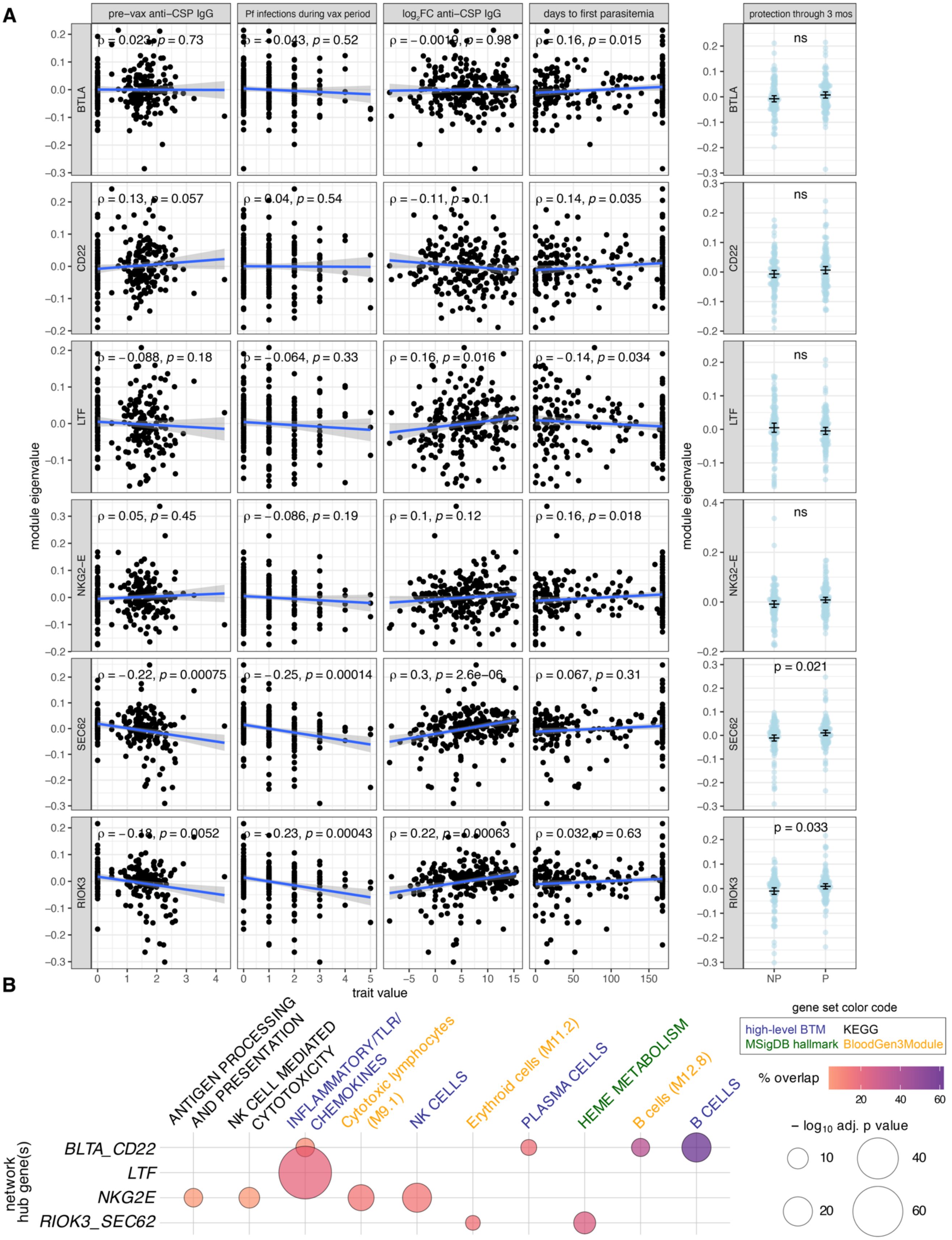

Figure S6. Relationship between WGCNA module eigenvalues and traits and module overrepresentation analysis. Related to Figure 4. **(A)** Scatter plots and box plots showing trends and distribution of module eigenvalue vs. trait relationships summarized in Figure 4B. **(B)** Overrepresentation analysis of genes within WGCNA-derived networks in Figure 4C using the indicated gene sets. Only pathways/modules with B-H adjusted p values < 0.01 (FDR<1%), minimum gene set size of 20, and overlap >5% are shown.

Table S4. Cox proportional hazards analysis of PfSPZ Vaccine-induced changes in *FSTL4* expression and time to first parasitemia in all vaccinated infants.

Infants from *all* PfSPZ Vaccine groups (n=155) were dichotomized as having expression upregulated or downregulated post-vaccination if $\log_2(CPM_{post\text{-}vax}/CPM_{baseline})$ >0 or <0, respectively.

Gene	Covariate	n	number of events	HR	LCI	UCI	P value	Significant
FSTL4	upregulated post-vax (ref:downregulated post-vax)	169	107	0.643	0.433	0.954	0.0282	*
FSTL4	gender (ref:female)	169	107	0.92	0.618	1.37	0.679	
FSTL4	Wagai (ref:Siaya)	169	107	0.629	0.421	0.939	0.0234	*
FSTL4	CSP-specific IgG baseline	169	107	1	0.795	1.26	0.999	
FSTL4	number of Pf infections during vaccination period	169	107	1.25	1.07	1.46	0.00465	**
FSTL4	$9.0x10^5$ PfSPZ (ref:$4.5x10^5$ PfSPZ)	169	107	1.1	0.693	1.76	0.677	
FSTL4	$1.8x10^6$ PfSPZ (ref:$4.5x10^5$ PfSPZ)	169	107	0.899	0.551	1.47	0.669	

Table S5. Spearman correlations between time to first parasitemia, post-vaccination CSP-specific IgG, transcriptomic features, and non-transcriptomic data.

Provided as a separate spreadsheet.

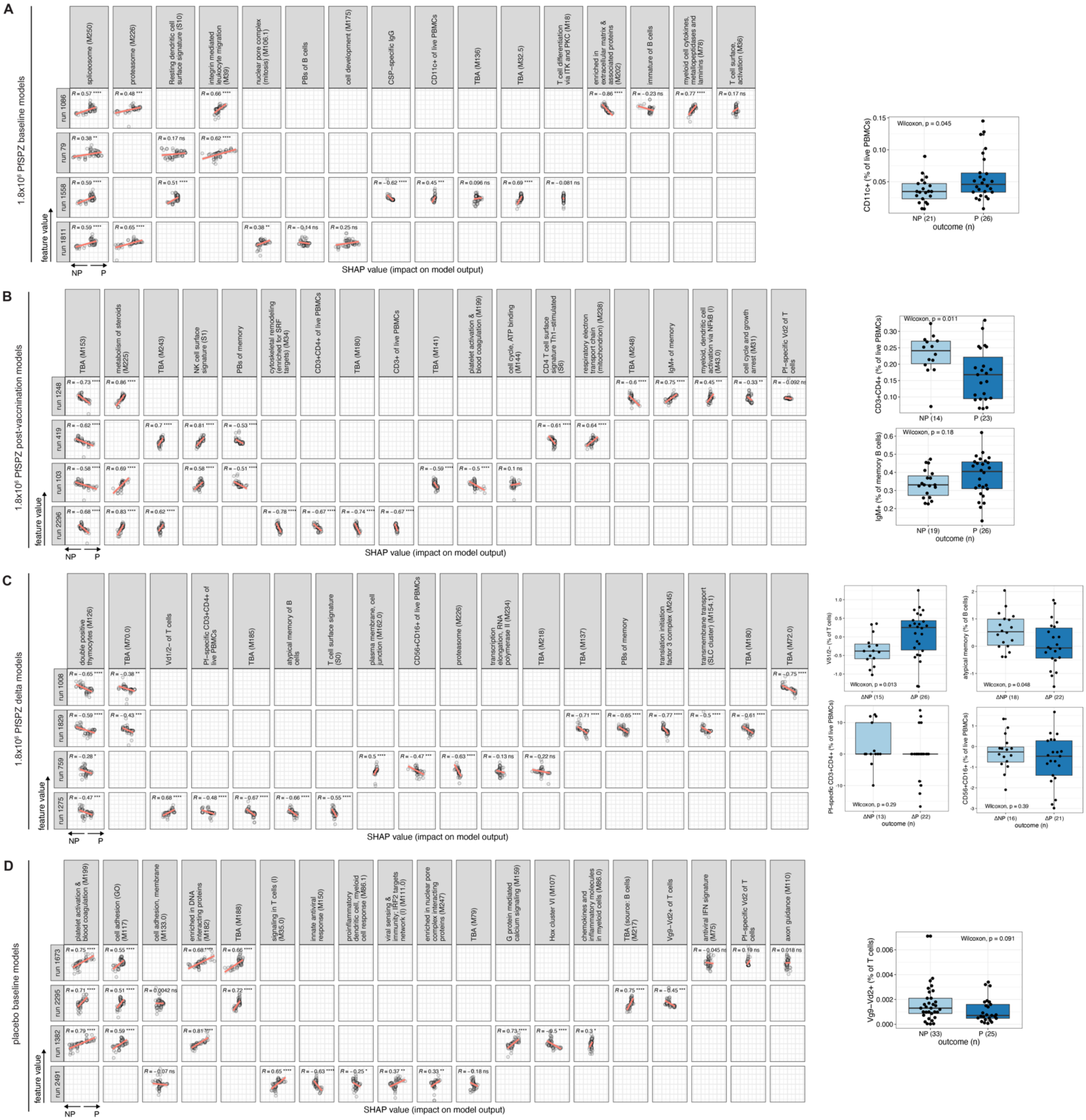

Figure S7. Correlations between feature values and SHAP values among 4 best performing models for each feature group.

Related to Figures 6 and 7. Scatter plots showing pairwise correlations for top 4 models based on composite performance metrics that include accuracy, area under the ROC curve, Brier score, kappa, and log-loss. Separate analyses were performed using gene expression, CSP-specific IgG, immunophenotyping (flow-cytometric), and cytokine features (baseline only) from **(A)** 1.8x10^6 PfSPZ, baseline; **(B)** post-vaccination; **(C)** 1.8x10^6 PfSPZ, delta (post-vaccination/baseline); and **(D)** placebo, baseline. Gene expression collapsed into either high-annotation level blood transcription modules (BTMs). For left panels, significance of correlations determined by Pearson's. *p<0.05, **p<0.01, ***p<0.001, ****p<0.0001, ns=not significant. Boxplots on right show NP vs. P differences in immunophenotypic features within the top 4 models, with significance determined by Wilcoxon test. SHAP = SHapley Additive exPlanations, P = protected, NP = not protected.

Table S6. Machine learning across-fold model performance metrics.

Provided as a separate spreadsheet.

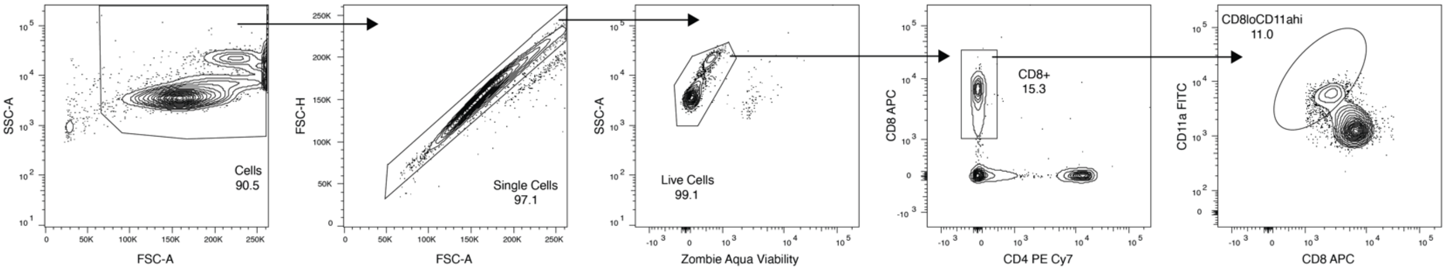

Figure S8. Gating strategies for RAS-induced CD8^{+} T cells.

Related to Figure 8. Representative flow cytometry plots identifying RAS-induced CD8^{+} T cells (CD8^{lo}CD11a^{hi}). Value within plot is the percent of all circulating CD8^{+} T cells that are CD8^{lo}CD11a^{hi}.

Table S7. Logistic regression model demonstrating significant three-way interaction between CSP-specific antibodies, CD14+ monocytes, and receipt of placebo/1.8x10^6 PfSPZ on protection.

**Characteristic**	**N**	**log(OR)**[1]	**95% CI**	**p**
log_{10}(CSP-specific IgG)	91	-2.2	-4.6, -0.49	**0.035**
CD14+ monocytes	91	-21	-54, 0.56	0.14
treat	91			
Placebo (reference)		—	—	
1.8x10^6 PfSPZ		-1.3	-4.9, 1.6	0.4
logCSPIgG * FACS_CD14+CD16-_of_live_monocytes	91	23	5.7, 47	**0.031**
logCSPIgG * treat	91			
logCSPIgG * 1.8x10^6 PfSPZ		2.4	0.34, 5.1	**0.042**
FACS_CD14+CD16-_of_live_monocytes * treat	91			
FACS_CD14+CD16-_of_live_monocytes * 1.8x10^6 PfSPZ		23	-2.3, 58	0.14
logCSPIgG * FACS_CD14+CD16-_of_live_monocytes * treat	91			
logCSPIgG * FACS_CD14+CD16-_of_live_monocytes * 1.8x10^6 PfSPZ		-29	-56, -9.5	**0.012**

[1]OR = Odds Ratio

Results are from a logistic regression model in which outcome (protected or not protected) is the dependent variable and log(CSP-specific IgG), CD14+ monocyte %, and treatment (1.8x106 PfSPZ or placebo) are independent variables. Model included a three-way interaction between these variables. Interactions are depicted by *. Show in bold are characteristics with p <0.05.

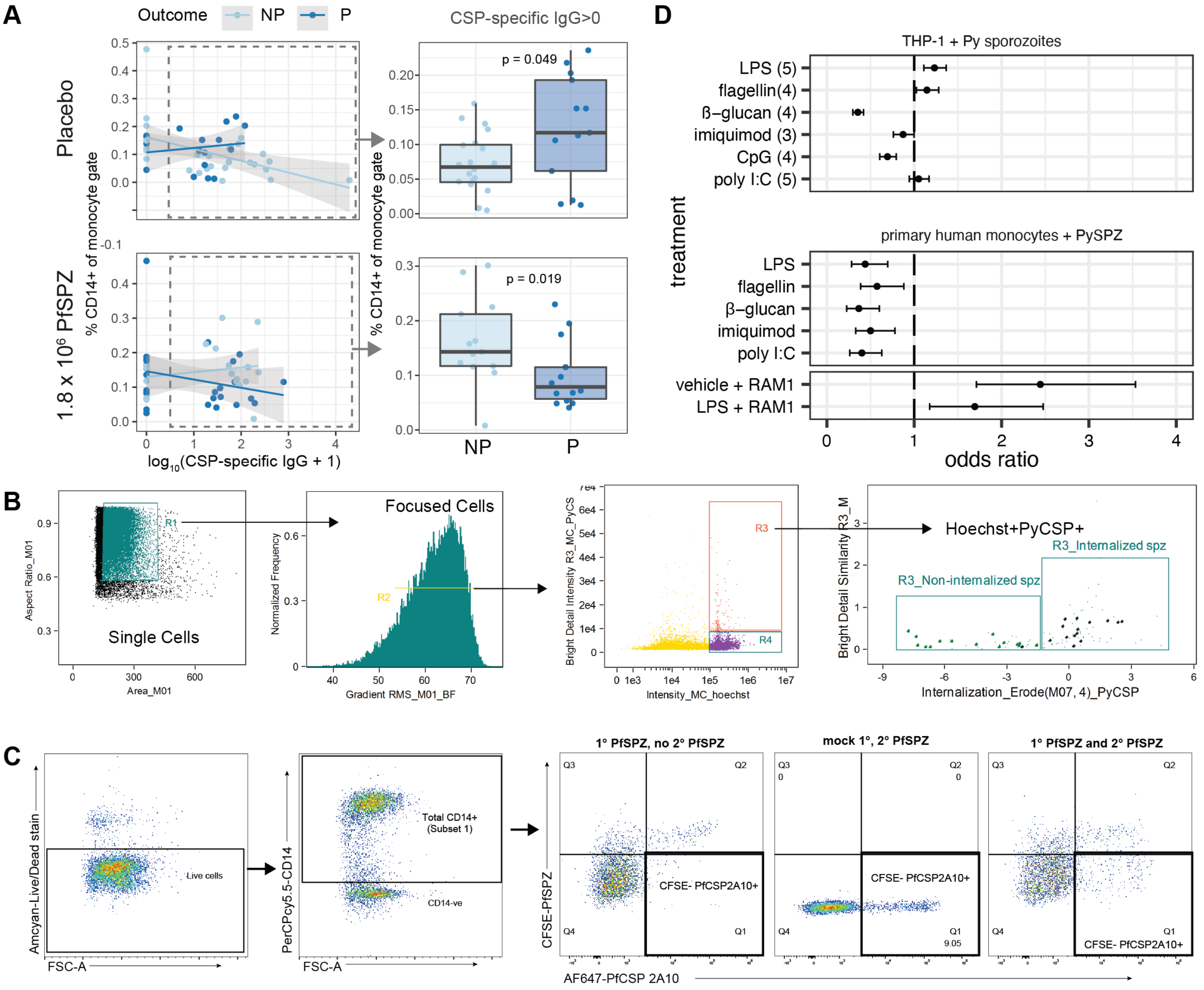

Figure S9. Detection of phagocytosed sporozoites, relationship between monocytes and CSP-specific IgG, and additional phagocytosis experiments.

Related to Figure 9. **(A)** % CD14+ peripheral monocytes by outcome and treatment for infants with baseline CSP-specific IgG>0 for infants who were not protected (NP) or protected (P) post-vaccination. **(B)** Gating strategy for identifying monocytes with internalized sporozoites by ImageStream. To accurately discriminate cells with cell-surface bound (external) sporozoites from cells with phagocytosed (internal) sporozoites within the Hoechst+PyCSP+ gate (R3), the "Feature Finder Wizard" was used to train two R3 populations by selecting cell images with external or internal sporozoites. A feature from default feature lists (e.g. location, shape, size, texture, signal strength) was then randomly selected to find features that best differentiated assigned populations. A comparison feature providing the best separation between the assigned populations was applied to all samples. **(C)** Gating strategy for identifying monocytes with internalized sporozoites by conventional flow cytometry. **(D)** Odds ratios with 95% confidence intervals for number of live human monocytes (THP-1 or primary peripheral blood) containing phagocytosed *P. yoelii* sporozoites (freshly dissected or purified, cryopreserved PySPZ) over all live monocytes for indicated treatment versus medium only control. Opsonization with anti-PyCSP RAM1 monoclonal antibody is shown as a positive control (bottom plot), where reference was isotype control antibody. Significance was determined by Fisher's exact test. For THP-1 experiments, data are combined from the number of experiments indicated in parentheses. For primary monocyte experiments, data represents two independent experiments.

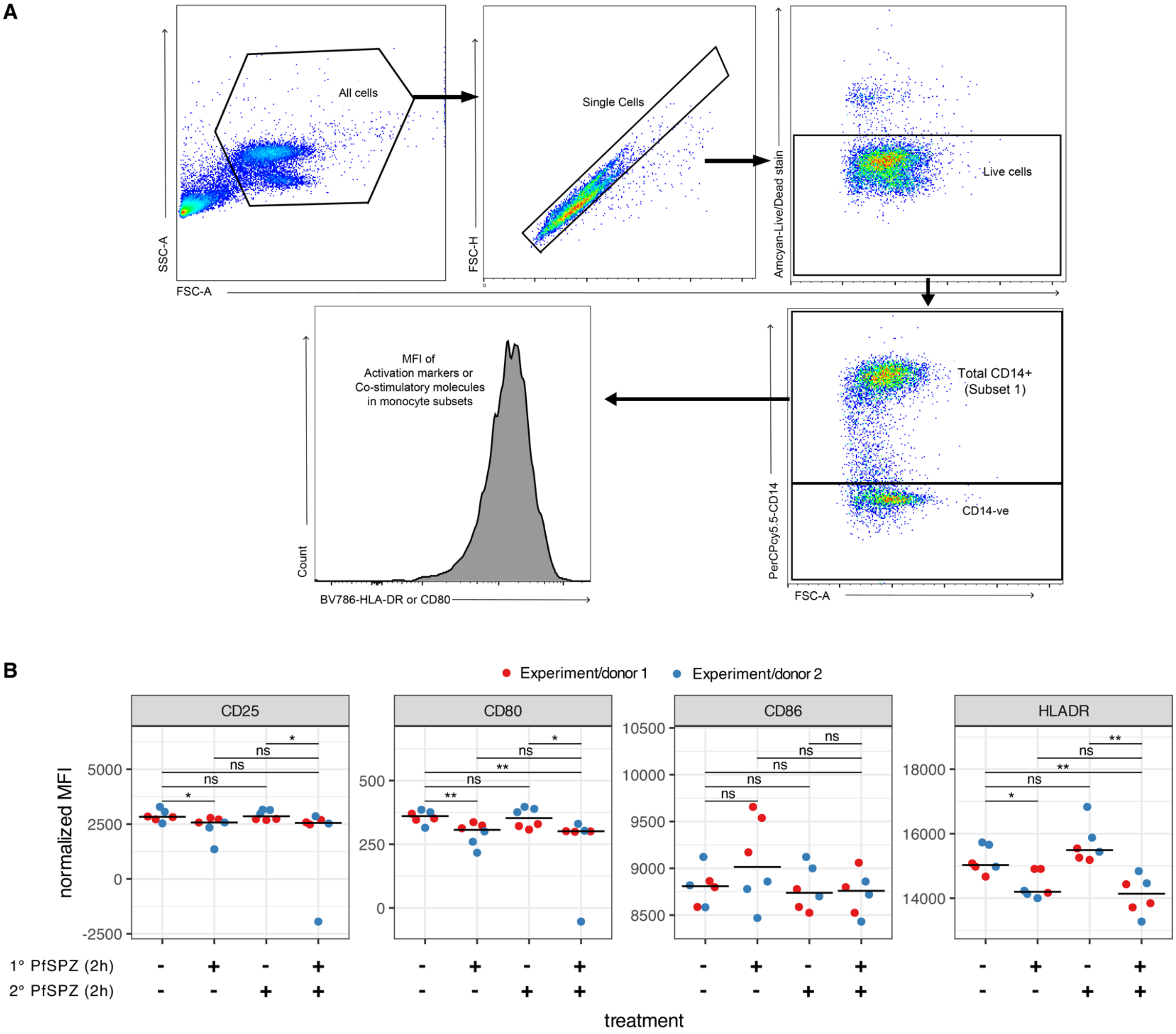

Figure S10. Pre-exposure to *P. falciparum* sporozoites (PfSPZ) decreases surface expression of activation markers in peripheral blood CD14+ monocytes freshly isolated from human donors.

Related to Figure 9. **(A)** Gating strategy for assessing surface expression of activation markers on CD14+ monocytes. **(B)** Surface expression of indicated monocyte activation markers after primary and secondary exposure to PfSPZ or media only. Points represent technical replicates and color represents independent experiments using different donors. Significance was determined by Wilcoxon test, with p values represented as * <0.05, ** <0.01, and ns = not significant. MFI = mean fluorescence intensity.

Table S8. Pre-vaccination baseline expression of innate-related low-annotation BTMs by CSP-specific IgG status at baseline in non-protected (NP) and protected (P) infants for Placebo and 1.8x10^6 PfSPZ Vaccine groups.

Provided as a separate spreadsheet.

Table S9. Resources Table

Provided as a separate spreadsheet.
