# Supplementary material for: Innate immune activation restricts priming and protective efficacy of the radiation-attenuated PfSPZ malaria vaccine
Source: JCI Insight. 2024 Apr 30;9(11):e167408. doi: 10.1172/jci.insight.167408 (PMC11382880; doi:10.1172/jci.insight.167408)
Supplement: Supplemental table 4 [file jciinsight-9-167408-s113.docx]

**Table S4. Cox proportional hazards analysis of PfSPZ Vaccine-induced changes in *FSTL4* expression and time to first parasitemia in all vaccinated infants.**

Infants from *all* PfSPZ Vaccine groups (n=155) were dichotomized as having expression upregulated or downregulated post-vaccination if log_2_(CPM_post-vax_/CPM_baseline_) >0 or <0, respectively.

| **Gene** | **Covariate** | **n** | **Number**  **of events** | **HR** | **LCI** | **UCI** | **P**  **value** | **Significant** |
| --- | --- | --- | --- | --- | --- | --- | --- | --- |
| FSTL4 | upregulated post-vax (ref:downregulated post-vax) | 169 | 107 | 0.643 | 0.433 | 0.954 | 0.0282 | * |
| FSTL4 | gender (ref:female) | 169 | 107 | 0.92 | 0.618 | 1.37 | 0.679 |  |
| FSTL4 | Wagai (ref:Siaya) | 169 | 107 | 0.629 | 0.421 | 0.939 | 0.0234 | * |
| FSTL4 | CSP-specific IgG baseline | 169 | 107 | 1 | 0.795 | 1.26 | 0.999 |  |
| FSTL4 | number of Pf infections during vaccination period | 169 | 107 | 1.25 | 1.07 | 1.46 | 0.00465 | ** |
| FSTL4 | 9.0x10^5^ PfSPZ (ref:4.5x10^5^ PfSPZ) | 169 | 107 | 1.1 | 0.693 | 1.76 | 0.677 |  |
| FSTL4 | 1.8x10^6^ PfSPZ (ref:4.5x10^5^ PfSPZ) | 169 | 107 | 0.899 | 0.551 | 1.47 | 0.669 |  |
