# Supplementary material for: Innate immune activation restricts priming and protective efficacy of the radiation-attenuated PfSPZ malaria vaccine
Source: JCI Insight. 2024 Apr 30;9(11):e167408. doi: 10.1172/jci.insight.167408 (PMC11382880; doi:10.1172/jci.insight.167408)
Supplement: Supplemental table 7 [file jciinsight-9-167408-s116.docx]

| **Characteristic** | **N** | **log(OR)**^1^ | **95% CI**^1^ | **p-value** |
| --- | --- | --- | --- | --- |
| log_10_(CSP-specific IgG) | 91 | -2.2 | -4.6, -0.49 | 0.035 |
| CD14+ monocytes | 91 | -21 | -54, 0.56 | 0.14 |
| treat | 91 |  |  |  |
| Placebo |  | — | — |  |
| 1.8 x 10^6 PfSPZ |  | -1.3 | -4.9, 1.6 | 0.4 |
| logCSPIgG * FACS_CD14+CD16-_of_live_monocytes | 91 | 23 | 5.7, 47 | 0.031 |
| logCSPIgG * treat | 91 |  |  |  |
| logCSPIgG * 1.8 x 10^6 PfSPZ |  | 2.4 | 0.34, 5.1 | 0.042 |
| FACS_CD14+CD16-_of_live_monocytes * treat | 91 |  |  |  |
| FACS_CD14+CD16-_of_live_monocytes * 1.8 x 10^6 PfSPZ |  | 23 | -2.3, 58 | 0.14 |
| logCSPIgG * FACS_CD14+CD16-_of_live_monocytes * treat | 91 |  |  |  |
| logCSPIgG * FACS_CD14+CD16-_of_live_monocytes * 1.8 x 10^6 PfSPZ |  | -29 | -56, -9.5 | 0.012 |
| ^1^OR = Odds Ratio, CI = Confidence Interval | | | | |
